# Acupuncture alleviates osteoarthritis pain by remodeling the DRG neuro-immune microenvironment via Piezo2-mediated macrophage polarization

**DOI:** 10.3389/fimmu.2026.1844125

**Published:** 2026-07-30

**Authors:** Tianran Tang, Dandan Liu, Binbin Liu, Gongzi Zhang, Zhifeng Zhao, Lihai Zhang, Tianyu Jiang

**Affiliations:** 1 Medical School of Chinese People’s Liberation Army (PLA), Beijing, China; 2 Department of Rehabilitation Medicine, The Second Medical Center & National Clinical Research Center for Geriatric Diseases, Chinese PLA General Hospital, Beijing, China; 3 Department of Health Sciences, The Second Medical Center & National Clinical Research Center for Geriatric Diseases, Chinese PLA General Hospital, Beijing, China; 4 Department of Orthopaedics, The Fourth Medical Center Chinese PLA General Hospital, Beijing, China; 5 Department of Rehabilitation Medicine, The First Medical Center, Chinese PLA General Hospital, Beijing, China; 6 Department of Hepatopancreatobiliary Surgery, The First Medical Center, Chinese PLA General Hospital, Beijing, China

**Keywords:** dorsal root ganglion, macrophage polarization, neuro-immune modulation, osteoarthritis, Piezo2

## Abstract

**Background:**

Osteoarthritis (OA) pain is predominantly characterized by mechanical allodynia, and dorsal root ganglion (DRG) neuro-immune crosstalk serves as a critical driver of peripheral sensitization. Acupuncture at ST36 produces robust analgesia, yet the mechanism by which peripheral mechanical stimulation translates into DRG immune regulation remains unclear. Here we investigated whether acupuncture alleviates OA pain by modulating Piezo2-dependent neuro-immune interactions.

**Methods:**

Monosodium iodoacetate (MIA) was injected into the knee joint to establish OA in mice. Manual acupuncture at ST36 was performed for 7 consecutive days. Pain behaviors, knee histopathology, and DRG immune profiles were assessed by von Frey test, OARSI scoring, flow cytometry, immunofluorescence, Western blot, Luminex assay, and RNA sequencing. Pharmacological inhibition (GsMTx4), GM-CSF neutralization, macrophage depletion, and AAV9-mediated Piezo2 activation in DRG neurons were used to verify causality.

**Results:**

Acupuncture significantly elevated mechanical paw withdrawal thresholds and attenuated cartilage destruction in OA mice. It did not alter systemic inflammatory cytokines but specifically normalized the DRG neuro-immune microenvironment: downregulating Piezo2 in knee-innervating DRG sensory neurons, reducing pro-inflammatory mediators including GM-CSF, and increasing the CD206-positive/M2-like macrophage phenotype. Inhibition of Piezo channels or neutralization of GM-CSF mimicked acupuncture analgesia, whereas DRG-specific Piezo2 activation recapitulated immune dysregulation and mechanical hypersensitivity. *In vitro*, conditioned medium from Piezo2-inhibited DRG neurons promoted a CD206-positive macrophage phenotype in bone marrow-derived macrophages.

**Conclusion:**

These findings reveal a novel neuro-immune mechanism underlying acupuncture analgesia. Acupuncture suppresses Piezo2 expression in DRG sensory neurons, reduces inflammatory mediators including GM-CSF, and reshapes the DRG immune microenvironment toward anti-inflammatory macrophage polarization. Our study identifies Piezo2 as a key mechanotransducer linking acupuncture to DRG neuro-immune modulation, providing a potential therapeutic target for OA pain.

## 1 Introduction

Osteoarthritis (OA) is the most prevalent degenerative joint disease and a leading cause of chronic pain and disability worldwide (1). Pain is the primary complaint driving clinical consultation, yet its management remains challenging due to complex peripheral and central sensitization mechanisms. Current pharmacotherapies such as non-steroidal anti-inflammatory drugs are limited by systemic side effects, highlighting an urgent need for safer, mechanism-based interventions (2–4).

Chronic OA pain is closely linked to low-grade inflammation and aberrant sensory signaling. The dorsal root ganglion (DRG) serves as the primary relay station for somatosensory transmission, where sensory neurons and immune cells form a tightly regulated microenvironment (5–7). Emerging evidence indicates that neuronal–immune crosstalk in the DRG promotes inflammatory mediator release, immune cell infiltration, and nociceptor hyperexcitability, thereby sustaining mechanical allodynia (8–10). Macrophages are among the most abundant immune cells in the DRG; their phenotypic balance between pro-inflammatory M1 and anti-inflammatory M2 states critically modulates neuroinflammation and pain progression. However, the upstream molecular regulators that govern DRG macrophage polarization in OA remain poorly defined.

Piezo2 is a mechanosensitive cation channel highly expressed in DRG sensory neurons, particularly in nociceptors and proprioceptors (11). Mounting evidence demonstrates that Piezo2 contributes to mechanical allodynia in inflammatory, neuropathic, and osteoarthritis pain conditions (11–13). Inflammatory mediators can enhance Piezo2-mediated mechanotransduction in DRG sensory neurons, and genetic deletion or suppression of Piezo2 attenuates inflammation-, nerve injury-, and osteoarthritis-associated mechanical hypersensitivity (14). Given that acupuncture is inherently a form of controlled mechanical stimulation applied to somatic acupoints (15–17), we hypothesized that acupuncture may modulate Piezo2 to interrupt pathological DRG neuro-immune crosstalk.

Although acupuncture has been reported to regulate macrophage polarization in inflammatory diseases (18, 19), the molecular connection between acupuncture-induced mechanical signals and DRG immune remodeling has not been established. In the present study, we demonstrate that manual acupuncture at ST36 remodels the DRG neuro-immune microenvironment to alleviate OA pain. Mechanistically, acupuncture downregulates Piezo2 in knee-innervating DRG neurons, reduces local inflammatory mediators including GM-CSF, and promotes a shift toward a CD206-positive/M2-like macrophage phenotype. Our findings define a Piezo2-mediated neuro-immune axis as a novel mechanism underlying acupuncture analgesia, offering a translational target for treating OA pain.

## 2 Materials and methods

### 2.1 Animals

Male C57BL/6 mice (6 weeks old) were purchased from Beijing Vital River Laboratory Animal Technology Co., Ltd. All mice were housed in a specific-pathogen-free (SPF) environment with a 12 h light/dark cycle, controlled temperature (22 ± 2 °C) and humidity (50% ± 5%), and free access to food and water. All animal experiments were approved by the Ethics Committee of the Chinese People’s Liberation Army General Hospital and performed in accordance with the ARRIVE guidelines 2.0 and the National Institutes of Health Guide for the Care and Use of Laboratory Animals.

Experimental grouping and sample size: Mice were randomly divided into 8 groups

Con group/MIA group/MIA + ST36 acupuncture group/MIA + GsMTx4 (Piezo2 inhibitor) group/MIA + anti-GM-CSF neutralizing antibody group/MIA + clodronate liposomes (macrophage depletion) group/AAV9-Ctr (control virus) group/AAV9-Piezo2 (Piezo2 activation) group

The experimenters were blinded to group allocation during all behavioral tests and data analyses. Data are presented as mean ± SEM from n = 3–6 independent biological replicates.

### 2.2 Osteoarthritis model induction

The monosodium iodoacetate (MIA)-induced OA model was established as previously described (20, 21). Mice were anesthetized with 1% pentobarbital sodium (50 mg/kg, i.p.). Then, 15 μL of 10 mg/mL MIA (MCE, HY-D0849) was injected into the right knee joint cavity. Mice in the Con group received an equal volume of sterile normal saline. Behavioral tests were performed at 3 weeks after MIA injection to confirm successful establishment of mechanical allodynia.

### 2.3 Acupuncture treatment

Manual acupuncture stimulation was applied at the ST36 (Zusanli) acupoint, located 5 mm below the capitulum fibulae on the right hind limb. Mice in the acupuncture group were fixed gently and received acupuncture with a disposable stainless steel needle (0.25 × 13 mm). The needle was inserted vertically to a depth of 3 mm and manipulated with lifting-thrusting and twirling (1–3 Hz, mild reinforcing-reducing method) for 15 min per session, once daily for 7 consecutive days. Mice in the non-acupuncture groups were placed in the same holder without needle insertion.

### 2.4 Behavioral tests

#### 2.4.1 Mechanical sensitivity test (von Frey test)

Mice were adapted to the testing environment for at least 60 min before testing. Mechanical withdrawal thresholds were measured using von Frey filaments (North Coast Medical, NC12775-99) with the up–down method. Filaments were applied vertically to the plantar surface of the right hind paw until buckling occurred. A positive response was defined as rapid paw withdrawal, licking, or flinching. Each paw was tested 3 times at 5-min intervals, and the average value was calculated as the paw withdrawal threshold (g). All tests were performed in a blinded manner.

#### 2.4.2 Knee swelling measurement

The transverse diameter of the right knee joint (mediolateral width at the most swollen site) was measured daily at a fixed time using a digital vernier caliper. Each joint was measured 3 consecutive times, and the mean value was recorded as the degree of swelling.

### 2.5 Pharmacological interventions

Piezo2 inhibition: GsMTx4 (MCE, HY-P1410) was dissolved in normal saline and administered intraperitoneally (100 μg/kg) twice a week for 2 consecutive weeks, starting at 2 weeks after MIA injection (22).

GM-CSF neutralization: Anti-mouse GM-CSF neutralizing antibody (Selleck, A2148) was injected via the tail vein (200 μL per mouse) twice a week for 2 weeks.

Macrophage depletion: Clodronate liposomes (Yeasen, 43007ES08) were administered intrathecally (10 μL per mouse) 3 times a week for 2 weeks to deplete DRG-resident macrophages. Control mice received an equal volume of PBS liposomes (23).

### 2.6 AAV9 microinjection into DRG

Recombinant AAV9-hSyn-Piezo2 and AAV9-hSyn-Ctr viruses were constructed and packaged by GeneChem Co., Ltd. (Shanghai, China). The viral titer was 5 × 10¹² vg/mL. Under anesthesia, a small incision was made to expose the ipsilateral/right L3–L5 DRGs, and 1 μL of AAV9 solution was injected into each DRG using a 30-gauge needle at a speed of 0.2 μL/min. Mice were used for experiments 3 weeks after viral injection to ensure sufficient expression.

### 2.7 Histology and immunofluorescence

Knee joints were harvested and fixed in 4% paraformaldehyde (PFA) for 48 h, decalcified in 10% EDTA (pH 7.4) for 21 days, dehydrated, and embedded in paraffin. Tissue sections (5 μm) were stained with hematoxylin and eosin (HE), Safranin O/Fast Green, and Toluidine Blue. Cartilage damage was evaluated using the OARSI score (Grade 0–6) (24). Coronal sections containing both femoral and tibial articular cartilage surfaces were selected for histological evaluation.

DRGs were fixed in 4% PFA for 4 h, cryoprotected in 30% sucrose, and sectioned at 20 μm. Sections were permeabilized with 0.3% Triton X-100, blocked with 5% normal goat serum, and incubated with primary antibodies overnight at 4 °C: Piezo2 (Proteintech, 15939-1-AP, 1:200)/CGRP (Abinvivo, B587301, 1:100)/F4/80 (Servicebio, GB113373, 1:500)/CD206 (Servicebio, GB113497, 1:400)/GM-CSF (Proteintech, 17762-1-AP, 1:400)/iNOS(Servicebio, GB11119, 1:100), as indicated in the corresponding experiments.

Fluorescent secondary antibodies were incubated for 1 h at room temperature. Images were captured using a confocal microscope, under identical acquisition settings for the groups being compared. For co-localization analysis, merged confocal images and enlarged regions of interest (ROIs) were used to evaluate the spatial relationship between Piezo2 and FG or CGRP signals. Line-scan intensity profiles were generated across representative FG-positive or CGRP-positive DRG neuronal regions to visualize signal overlap. Quantitative analysis was performed using ImageJ. Piezo2 fluorescence intensity was measured within FG-positive or CGRP-positive neuronal regions and normalized to the corresponding ROI area. The analysis focused on fluorescence intensity within marker-positive regions rather than the proportion of double-positive cells.

### 2.8 Neural tracing

Fluoro-Gold (FG, Sigma, 39286) was prepared as a 2% solution. Ten microliters of FG was injected into the right knee joint at 7 days after MIA administration. Seven days after FG injection, ipsilateral/right L3–L5 DRGs were harvested, fixed, cryoprotected, and sectioned as described above. FG fluorescence was detected using the DAPI channel of the confocal microscope (25). FG-labeled cells in L3–L5 DRGs were used to identify knee-innervating DRG neurons. For co-localization assessment, FG fluorescence was examined together with Piezo2 immunofluorescence in the same DRG sections, and Piezo2 fluorescence intensity was quantified within FG-positive neuronal regions using ImageJ.

### 2.9 Western blotting

After euthanasia, the ipsilateral right L3–L5 dorsal root ganglia corresponding to the MIA-injected knee were rapidly dissected and processed for subsequent analyses. DRG tissues were lysed in RIPA buffer containing protease and phosphatase inhibitors. Equal amounts of protein (15 μg) were separated by 12% SDS-PAGE and transferred to PVDF membranes. Membranes were blocked with 5% BSA and incubated with primary antibody against Piezo2 (Proteintech, 15939-1-AP, 1:1000) overnight at 4 °C, followed by HRP-conjugated secondary antibody. Protein bands were visualized using ECL reagent, and band intensity was quantified using ImageJ, normalized to β-actin.

### 2.10 Flow cytometry

Ipsilateral/right DRGs (L3–L5) were dissected and digested with collagenase IV and DNase I to prepare single-cell suspensions. Cells were stained with fluorophore-conjugated antibodies: CD45, CD11b, F4/80, Ly6G, Ly6C, CD206. Flow cytometry was performed on a BD FACSCanto II system, and data were analyzed using FlowJo software. For each sample, at least 20,000 cells were recorded.

### 2.11 Luminex assay and ELISA

Ipsilateral L3–L5 DRG tissues were collected for cytokine detection. Because of the limited amount of mouse DRG tissue, lysates were prepared using a standardized tissue-to-buffer ratio to minimize sample-loading variation. Briefly, 200 μL of PBS-based lysis buffer was added per 10 mg of DRG tissue. Samples were homogenized using a tissue homogenizer, incubated at 4 °C for 20 min, and centrifuged at 13,000 rpm for 10 min at 4 °C. The supernatants were collected and used for subsequent Luminex and ELISA analyses.

The levels of multiple cytokines (IL-6, TNF-α, GM-CSF, CGRP, etc.) were detected using a Luminex multiplex assay kit (Bio-Rad, 12009159) and commercial ELISA kits (Kelu Biotechnology) according to the manufacturers’ protocols. For DRG tissue lysates, cytokine concentrations were normalized to the wet weight of the corresponding DRG tissue and expressed as pg/mg tissue. The normalized cytokine level was calculated as follows:

normalized cytokine level = cytokine concentration in lysate supernatant (pg/mL) × lysate volume (mL)/DRG tissue weight (mg).

For serum ELISA, blood was collected from deeply anesthetized mice by retro-orbital bleeding at the indicated endpoint. Samples were allowed to clot for 15 min at room temperature and centrifuged at 1000g for 15 min at 4 °C. Serum was aliquoted and stored at −80 °C until ELISA. Samples were diluted according to the manufacturers’ instructions.

### 2.12 RNA sequencing and bioinformatics analysis

Total RNA was extracted from ipsilateral/right L3–L5 DRGs (2 mice per biological replicate). RNA sequencing was performed on an Illumina platform. Differentially expressed genes (DEGs) were identified with thresholds of |log2FC| ≥ 0.5 and padj ≤ 0.05 (26). GO and KEGG enrichment analyses were performed using the clusterProfiler package in R.

### 2.13 Neuron-macrophage co-culture and conditioned medium

Primary DRG neurons were isolated from neonatal C57BL/6 mice and cultured in neurobasal medium with B-27 supplement. For conditioned medium (CM) collection, neurons were stimulated with TNF-α (20 ng/mL) for 24 h, followed by GsMTx4 (10 ng/mL) for 3 days. Bone marrow-derived macrophages (BMDMs) were incubated with neuronal CM for 24 h.

### 2.14 Statistical analysis

All data are presented as mean ± standard error of the mean (SEM). Statistical analyses were performed using GraphPad Prism 7.0 and R software (version 26.0).

Comparisons between two groups: Student’s t-test.

Comparisons among three or more groups: One-way ANOVA followed by Tukey’s *post hoc* test.

Behavioral data with multiple time points: repeated-measures two-way ANOVA followed by Bonferroni *post-hoc* tests.

P < 0.05 was considered statistically significant (*P < 0.05, **P < 0.01, ***P < 0.001, ****P < 0.0001).

## 3 Results

### 3.1 Manual acupuncture at ST36 alleviates mechanical allodynia and protects articular cartilage in MIA-induced OA mice

**Figure 1:**
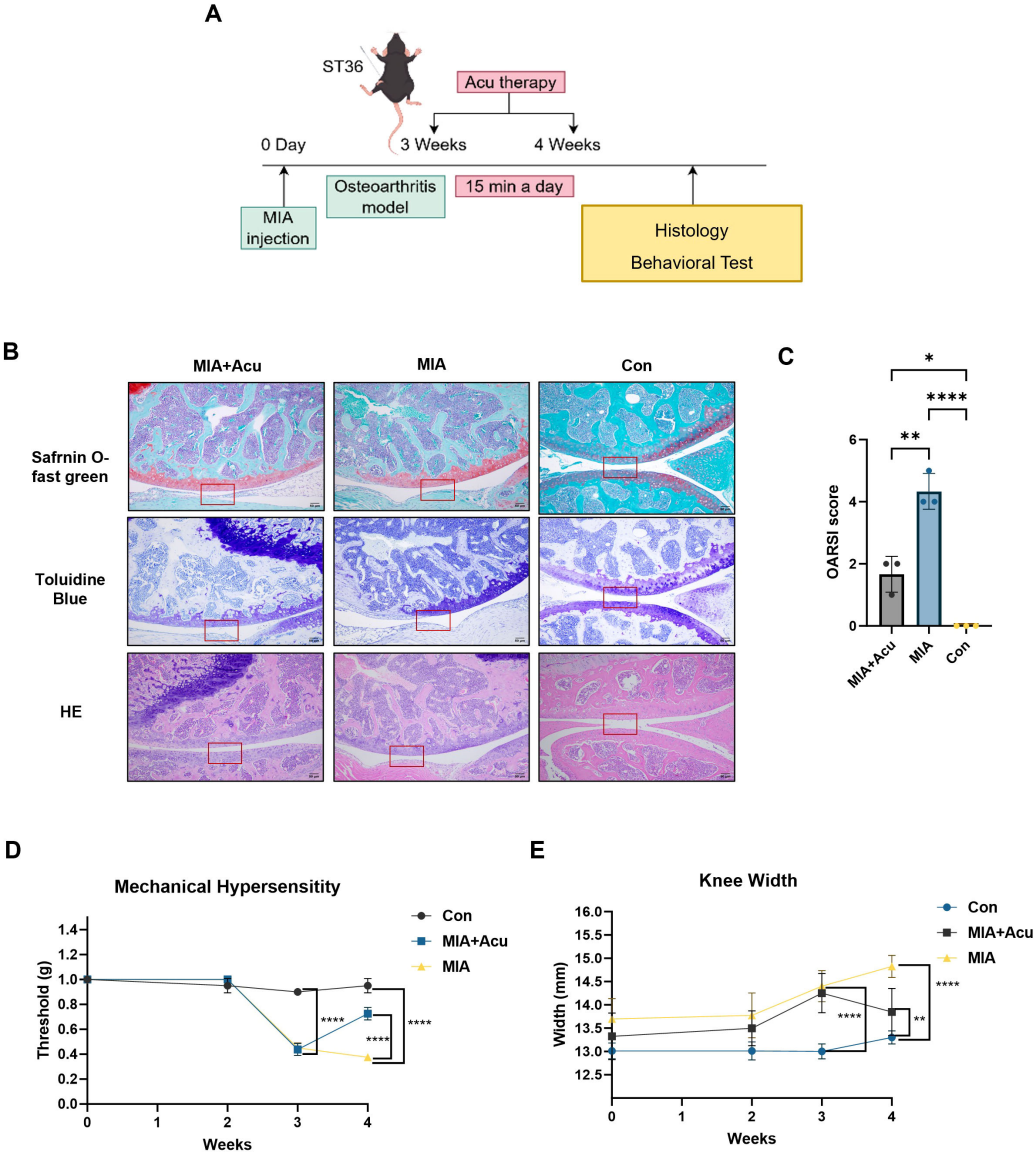
Manual acupuncture at ST36 alleviates mechanical allodynia and protects articular cartilage in MIA-induced OA mice. **(A)** Schematic diagram of the experimental workflow. Mice were intra-articularly injected with MIA to establish an osteoarthritis (OA) model. At 3 weeks post-injection, manual acupuncture (Acu) was applied daily at the ST36 acupoint for 15 minutes over a consecutive 7-day period. Pain behavioral tests and histopathological analyses were subsequently performed. **(B)** Representative images of Safranin O-fast green, Toluidine Blue, and Hematoxylin & Eosin (HE) staining of knee joint sections from the Control (Con), MIA, and MIA+Acu groups. Red boxes indicate representative regions of articular cartilage used to illustrate cartilage degeneration and matrix loss. Scale bars represent 100 μm. **(C)** Quantitative analysis of the OARSI score based on Safranin O-fast green staining. Data are presented as mean ± SEM (n = 6). **(D)** Time-course changes in mechanical withdrawal thresholds measured by the von Frey test. **(E)** Time-course changes in knee joint width measured using a digital vernier caliper. Statistical significance was determined by two-way repeated-measures ANOVA followed by Bonferroni *post-hoc* test for **(D, E)**, and one-way ANOVA for **(C)**. The schematic in panel A was created using FigDraw.

To evaluate the analgesic and chondroprotective effects of acupuncture, we established a mouse OA model by intra-articular injection of monosodium iodoacetate (MIA). Three weeks after modeling, mice developed stable mechanical allodynia and obvious cartilage damage (**Supplementary Figures 1A, B**). Manual acupuncture at ST36 was performed for 15 min daily for 7 consecutive days with standardized lifting-thrusting and twirling manipulation (**Figure 1A**).

Histological staining (H&E, Safranin O/Fast Green, Toluidine Blue) and OARSI scoring revealed that ST36 acupuncture markedly attenuated MIA-induced cartilage erosion and matrix loss (**Figures 1B, C**). Consistently, von Frey testing showed that acupuncture significantly elevated paw withdrawal mechanical thresholds compared with the MIA group (**Figure 1D**). Meanwhile, the transverse diameter of the swollen knee joint was notably reduced after acupuncture treatment (**Figure 1E**). These results confirm that ST36 acupuncture exerts robust analgesic and chondroprotective effects in OA mice.

### 3.2 Acupuncture reshapes the local neuro-immune microenvironment in DRGs without altering systemic inflammation

**Figure 2:**
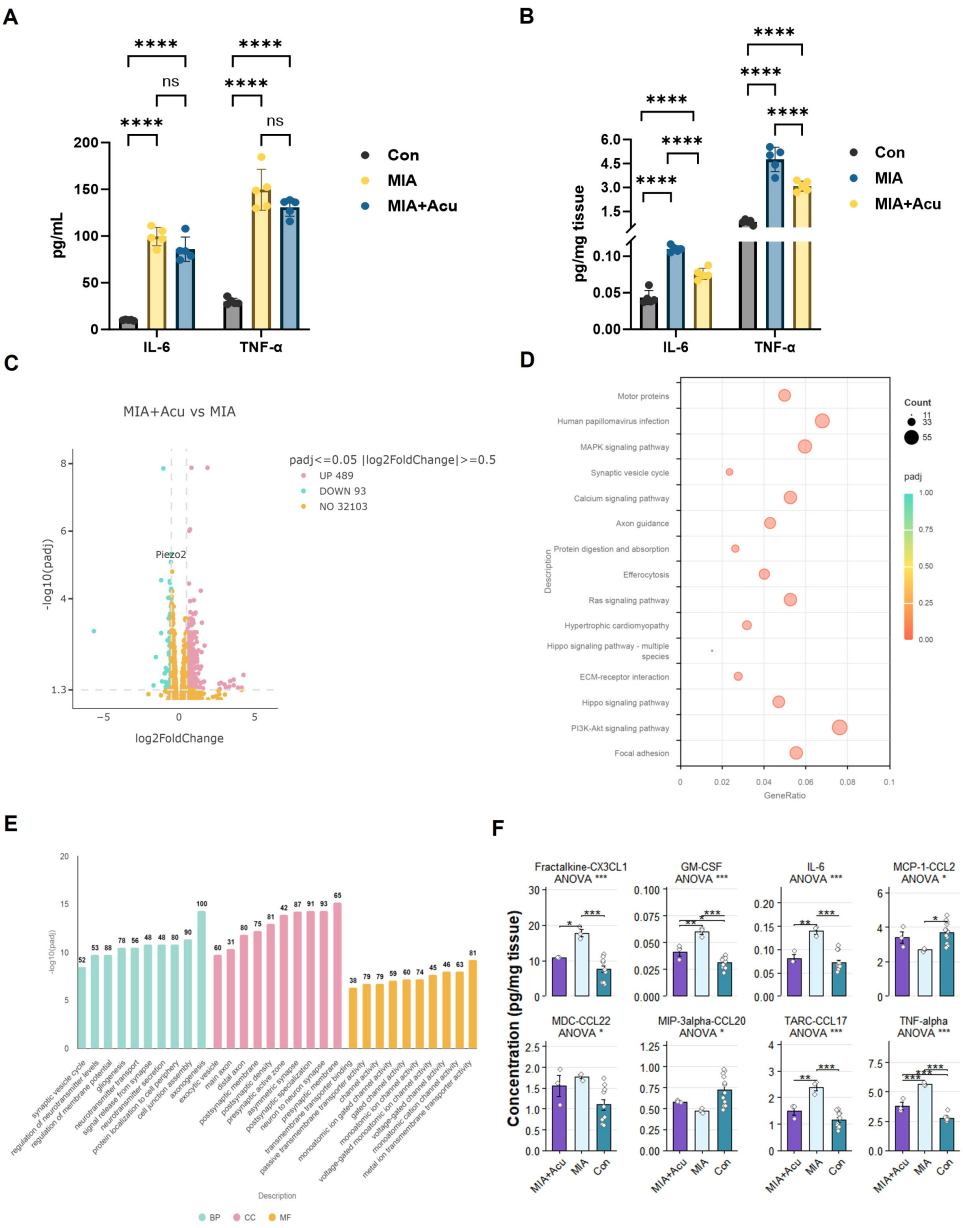
Acupuncture reshapes the local neuro-immune microenvironment in DRGs without altering systemic inflammation. **(A, B)** The concentrations of interleukin-6 (IL-6) and tumor necrosis factor-α (TNF-α) in serum and DRG tissue lysates were measured using an enzyme-linked immunosorbent assay (ELISA) in the control group (Con), the MIA group, and the MIA + acupuncture (Acu) group. **(C)** Volcano plot showing differentially expressed genes (DEGs) in L3-L5 dorsal root ganglia (DRGs) between MIA and MIA+Acu mice. DEGs were filtered with thresholds of padj < 0.05 and |log2FoldChange| ≥ 0.5. Red dots represent upregulated genes, blue dots represent downregulated genes, and gray dots represent non-differentially expressed genes. **(D)** GeneRatio enrichment plot of Kyoto Encyclopedia of Genes and Genomes (KEGG) pathway analysis for DEGs. The color gradient indicates adjusted *P* value (padj), and the dot size represents the number of genes enriched in each pathway. **(E)** Bar plot showing the top 20 enriched Gene Ontology (GO) terms categorized into biological process (BP), cellular component (CC), and molecular function (MF). The color gradient represents adjusted *P* value. **(F)** Multiplex Luminex assay profiling of key inflammatory cytokines in DRG tissue lysates. Data are presented as mean ± SEM (n = 5–6 per group). Statistical significance in **(A, B, F)** was determined by one-way ANOVA followed by Tukey’s *post-hoc* test. *P < 0.05, **P < 0.01, ***P < 0.001, ****P < 0.0001 vs. MIA group. The schematic in panel A was created using FigDraw.

We next examined whether acupuncture modulates systemic or regional inflammation. ELISA analysis of serum samples showed no significant changes in IL-6 and TNF-α levels after acupuncture, indicating that acupuncture does not induce widespread systemic immune alterations (**Figure 2A**).

Given the critical role of the dorsal root ganglion (DRG) in somatosensory transduction and peripheral sensitization, we focused on L3–L5 DRGs, which were confirmed by neural tracing to receive primary sensory innervation from the knee joint (**Supplementary Figure 1C**). In contrast to serum, DRG levels of IL-6 and TNF-α were significantly decreased by acupuncture (**Figure 2B**), suggesting that acupuncture exerts targeted immunomodulation within the DRG neuro-immune niche. To further define the molecular programs underlying this local DRG immunomodulation, we performed RNA sequencing of ipsilateral L3–L5 DRGs from MIA and MIA+Acu mice.

RNA-sequencing analysis identified 582 differentially expressed genes (DEGs) in DRGs between the MIA and acupuncture groups (|log2FC| ≥ 0.5, padj ≤ 0.05), with 489 upregulated and 93 downregulated (**Figure 2C**; **Supplementary Table 1**). GO enrichment highlighted terms related to transmembrane transporter activity, ion channel activity, and membrane-associated signaling. These terms are functionally related to sensory transduction but were not labeled as “mechanosensation” in the GO plot. KEGG analysis revealed prominent enrichment in extracellular matrix–receptor interaction, focal adhesion, PI3K-Akt, and MAPK signaling pathways, as well as efferocytosis (**Figures 2D, E**).

Multiplex Luminex assay further confirmed that acupuncture broadly suppressed DRG inflammatory mediators. Among 8 altered cytokines, IL-6, TNF-α, and GM-CSF were markedly reduced (**Figure 2F**; **Supplementary Table 2**). Together, these data demonstrate that ST36 acupuncture remodels the DRG local neuro-immune microenvironment rather than acting systemically.

### 3.3 Acupuncture promotes an anti-inflammatory macrophage phenotype in MIA-induced DRGs

**Figure 3:**
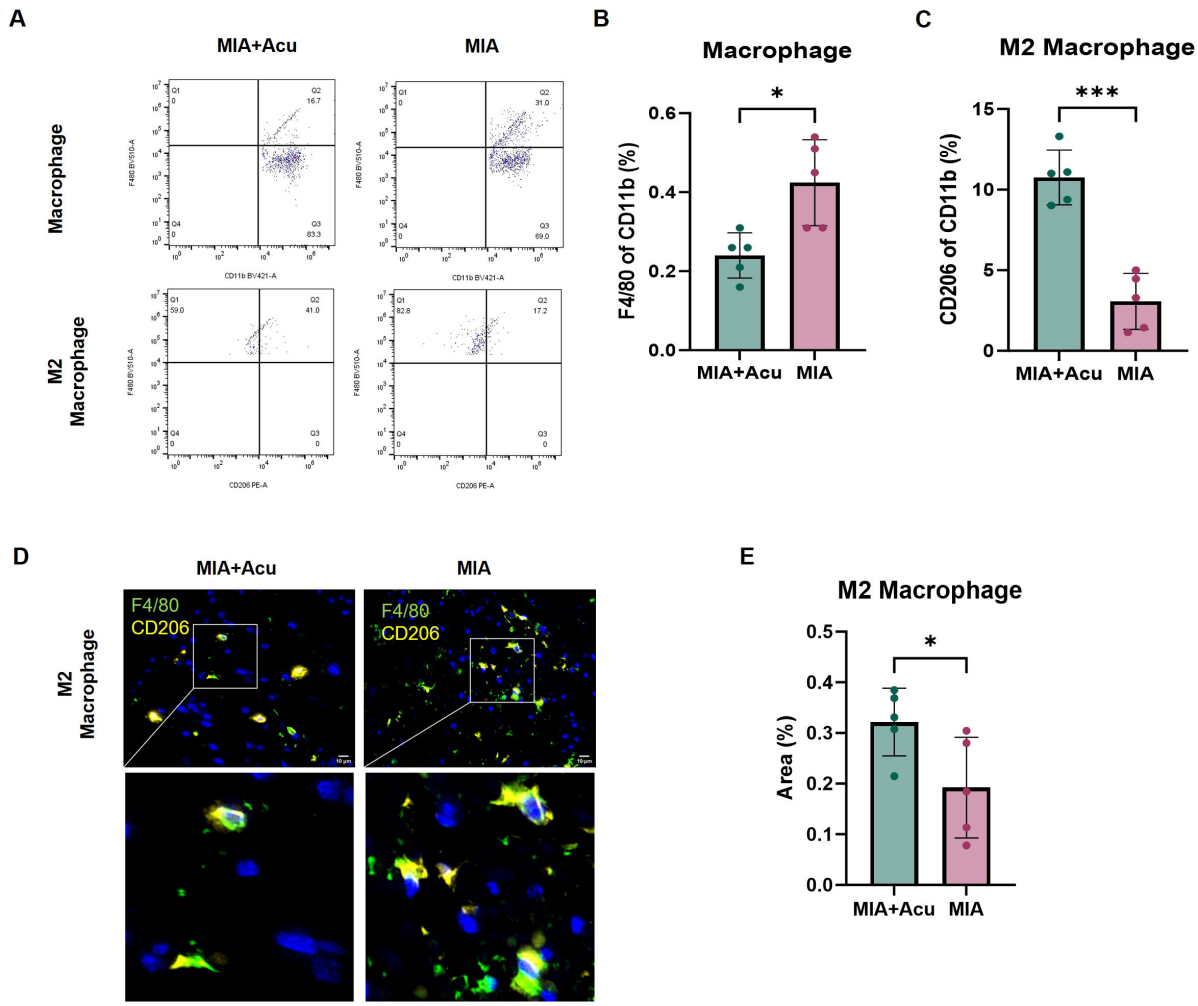
Acupuncture promotes an anti-inflammatory macrophage phenotype in MIA-induced DRGs. **(A)** Representative flow cytometry plots showing the gating strategy for macrophages (F4/80^+^CD11b^+^) and M2 macrophages (F4/80^+^CD206^+^) in isolated L3-L5 dorsal root ganglia (DRGs) from MIA and MIA+Acupuncture (Acu) mice. **(B, C)** Quantitative analysis of the percentage of F4/80^+^CD11b^+^ macrophages **(B)** and F4/80^+^CD206^+^ M2 macrophages **(C)** in the DRGs. **(D)** Representative immunofluorescence images of F4/80 (green) and CD206 (red) co-staining in DRG sections. Nuclei were counterstained with DAPI (blue). Scale bars represent 50 μm (upper panel) and 20 μm (lower panel). **(E)** Quantitative analysis of the CD206^+^ area percentage in immunofluorescence images. Data are presented as mean ± SEM (n = 5–6 per group). Statistical significance was determined by unpaired Student’s t-test. *P < 0.05, ***P < 0.001 vs. MIA group. The schematic in panel A was created using FigDraw.

Given the prominent immune-related signatures in transcriptomic data, we analyzed immune cell subpopulations in DRGs using flow cytometry. The detailed gating strategy is shown in **Supplementary Figure 1D**. Compared with the MIA group, acupuncture significantly reduced the total proportion of macrophages while increasing the percentage of anti-inflammatory M2 macrophages (**Figures 3A–C**). Acupuncture also decreased the abundance of neutrophils and pro-inflammatory Ly6C monocytes in DRGs (**Supplementary Figures 1E, F**).

Consistent with the flow-cytometric findings, immunofluorescent staining for F4/80, a pan-macrophage marker, and CD206, an M2-associated marker, showed an increased CD206-positive macrophage phenotype in the MIA + acupuncture group compared with the MIA group (**Figures 3D, E**). Representative Control DRG sections showed minimal or no clearly detectable F4/80/CD206-positive signals under the present staining conditions and are provided as baseline reference images in **Supplementary Figure 2A**.

To further assess whether acupuncture affected M1-like macrophages, we additionally performed immunofluorescent staining for F4/80 and iNOS. However, iNOS-positive F4/80-positive macrophages were barely detectable in DRG sections under the present staining conditions, and the signal was not sufficiently robust for reliable quantitative comparison among the Con, MIA, and MIA + acupuncture groups (**Supplementary Figure 2B**). Therefore, we did not draw a definitive conclusion regarding M1-like macrophage changes in the present study.

Together, these results suggest that ST36 acupuncture remodels the OA-associated DRG immune microenvironment by reducing inflammatory myeloid cell populations and shifting macrophages toward an anti-inflammatory M2-like phenotype.

### 3.4 Acupuncture downregulates Piezo2 expression in knee-innervating DRG neurons

**Figure 4:**
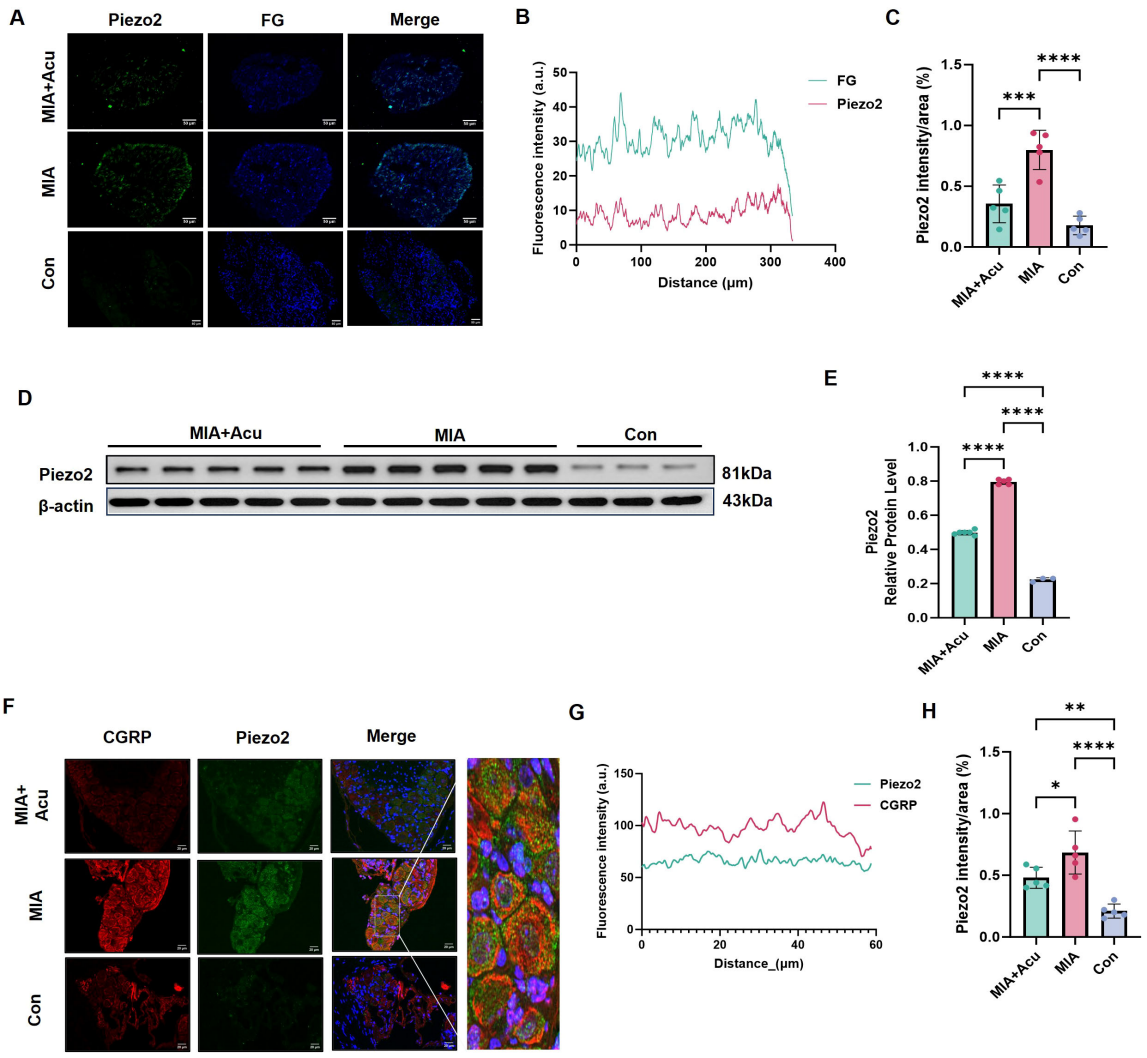
Acupuncture downregulates Piezo2 expression in knee-innervating DRG neurons. **(A)** Representative immunofluorescence images showing Piezo2 (green) expression in ipsilateral L3–L5 dorsal root ganglia (DRGs) retrogradely labeled with Fluoro-Gold (FG, blue) after knee-joint injection in the Con, MIA, and MIA + Acupuncture (Acu) groups. Merged images illustrate the spatial relationship between Piezo2 signals and FG-labeled knee-innervating DRG neurons. Scale bars, 50 μm. **(B)** Representative line-scan intensity profiles showing the spatial distribution of Piezo2 and FG fluorescence signals across selected FG-labeled DRG neuronal regions. **(C)** Quantitative analysis of Piezo2 fluorescence intensity within FG-positive neuronal regions. **(D)** Western blot analysis of Piezo2 protein expression in L3–L5 DRG lysates from the Con, MIA, and MIA + Acu groups. β-actin was used as the loading control. **(E)** Densitometric quantification of Piezo2 protein levels normalized to β-actin. **(F)** Representative immunofluorescence images showing CGRP (red), Piezo2 (green), and DAPI-stained nuclei (blue) in ipsilateral L3–L5 dorsal root ganglion sections from the Control (Con), MIA, and MIA + acupuncture (Acu) groups. Separate CGRP and Piezo2 channels and the corresponding merged images are shown. Enlarged images show the spatial relationship between Piezo2 and CGRP signals in MIA group. Scale bars, 20 μm. **(G)** Representative fluorescence-intensity profiles of CGRP and Piezo2 across the enlarged region shown in **(F)** Fluorescence intensity was plotted as a function of distance across the selected region. Partially overlapping fluorescence patterns indicate spatial overlap between CGRP and Piezo2 signals within the representative DRG neuronal region. The x-axis represents distance across the selected region, and the y-axis represents fluorescence intensity in arbitrary units. **(H)** Quantitative analysis of Piezo2 fluorescence intensity within CGRP-positive neuronal regions. Data are presented as mean ± SEM (n = 5–6 per group). Statistical significance was determined by one-way ANOVA followed by Tukey’s *post hoc* test. *P < 0.05, **P < 0.01, *** P < 0.001, ****P < 0.0001. The schematic in panel A was created using FigDraw.

As a key mechanotransducer implicated in mechanical allodynia, Piezo2 was hypothesized to link acupuncture stimulation to DRG neuro-immune regulation. Fluoro-Gold (FG) retrograde tracing after knee-joint injection revealed FG-labeled cells in ipsilateral L3–L5 DRGs, confirming the presence of knee-innervating DRG neurons. Immunofluorescence analysis showed that Piezo2 was detected in FG-labeled neuronal regions, and acupuncture reduced Piezo2 fluorescence intensity within FG-positive regions compared with the MIA group (**Figures 4A–C**).

Consistently, Western blot analysis confirmed that acupuncture markedly reduced Piezo2 protein expression in L3–L5 DRGs compared with the MIA group (**Figures 4D, E**). To further examine the spatial relationship between Piezo2 and the nociceptive neuronal marker CGRP, we performed Piezo2/CGRP double immunofluorescence staining. CGRP-positive neuronal profiles were readily detectable in the Control, MIA, and MIA + acupuncture groups (**Figure 4F**). A higher-magnification view of a representative MIA-group region showed partial spatial overlap between Piezo2 and CGRP signals within a subset of DRG neuronal profiles (**Figure 4G**). Quantification showed that acupuncture significantly reduced Piezo2 fluorescence intensity within CGRP-positive neuronal regions (**Figure 4H**). Together, these findings indicate that acupuncture downregulates Piezo2 expression in knee-innervating DRG sensory neurons, with Piezo2 signals also detected within a subset of CGRP-positive neuronal regions. This reduction may contribute to the attenuation of mechanical hypersensitivity.

### 3.5 GM-CSF is upregulated in MIA-induced DRGs and suppressed by acupuncture

**Figure 5:**
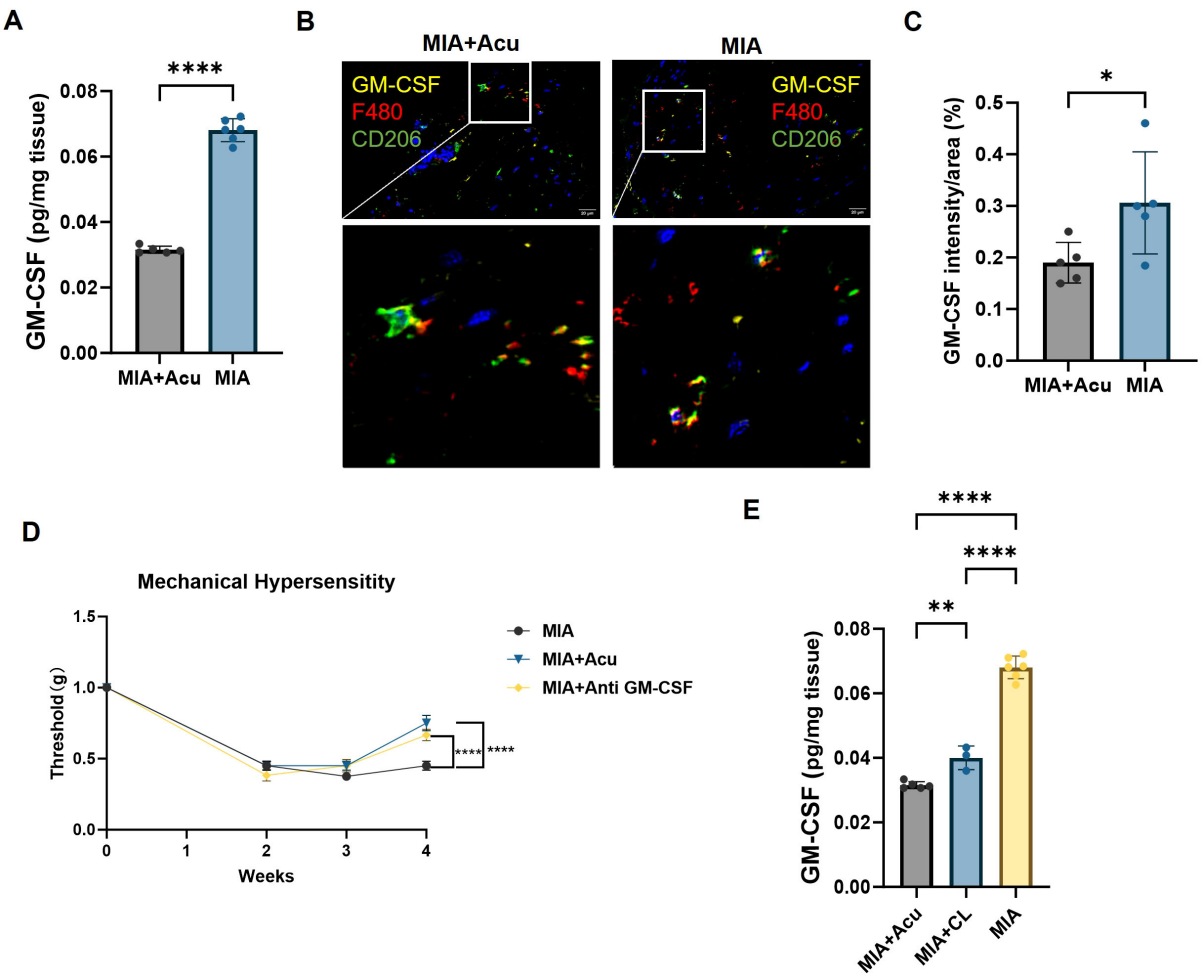
GM-CSF is upregulated in MIA-induced DRGs and suppressed by acupuncture. **(A)** ELISA quantification of granulocyte-macrophage colony-stimulating factor (GM-CSF) levels in ipsilateral L3–L5 DRG tissue lysates from the indicated groups. **(B)** Representative immunofluorescence images showing GM-CSF (yellow), F4/80 (red), and CD206 (green) staining in DRG sections from the MIA and MIA + Acu groups. Nuclei were counterstained with DAPI (blue). Enlarged images show representative regions of GM-CSF-positive immunoreactivity. Scale bars, 20 μm. **(C)** Quantitative analysis of GM-CSF fluorescence intensity in DRG sections. **(D)** Time-course changes in mechanical withdrawal thresholds measured by the von Frey test in the indicated groups following GM-CSF neutralization. **(E)** Quantitative analysis of DRG GM-CSF levels in the indicated treatment groups. Data are presented as mean ± SEM (n = 5–6 per group unless otherwise indicated). Statistical significance was determined by unpaired Student’s t-test for two-group comparisons, one-way ANOVA followed by Tukey’s *post hoc* test for multiple-group comparisons, and repeated-measures two-way ANOVA followed by Bonferroni *post hoc* test for behavioral data. The schematic in panel A was created using FigDraw.

Among the downregulated cytokines identified by Luminex analysis, GM-CSF was selected for further validation because of its established roles in myeloid-cell activation and inflammatory/arthritic pain (27–29). ELISA analysis showed that basal GM-CSF levels were low in Control DRGs, increased after MIA treatment, and were significantly reduced after acupuncture treatment (**Figure 5A**; **Supplementary Figure 3A**). Consistently, immunofluorescence staining showed weak GM-CSF signals in Control DRG sections, whereas MIA induced marked GM-CSF immunoreactivity that was attenuated by acupuncture (**Figures 5B, C**; **Supplementary Figure 3B**). These results indicate that GM-CSF is upregulated in the DRG during MIA-induced OA pain and is suppressed by acupuncture.

To validate the functional relevance of GM-CSF to OA pain, mice were treated with an anti-GM-CSF neutralizing antibody (**Supplementary Figure 3A**). Neutralization of GM-CSF significantly alleviated MIA-induced mechanical allodynia (**Figure 5D**). Similarly, macrophage depletion using clodronate liposomes reduced pain hypersensitivity and decreased DRG GM-CSF levels (**Figure 5E**; **Supplementary Figures 3C, D**). These results indicate that GM-CSF contributes to the maintenance of OA-related pain and represents one component of the inflammatory DRG microenvironment. Because macrophage phenotypes after GM-CSF neutralization were not directly examined in the present study, we avoided implying that GM-CSF directly mediates macrophage polarization.

### 3.6 Piezo2 inhibition attenuates OA pain and remodels the DRG immune microenvironment

**Figure 6:**
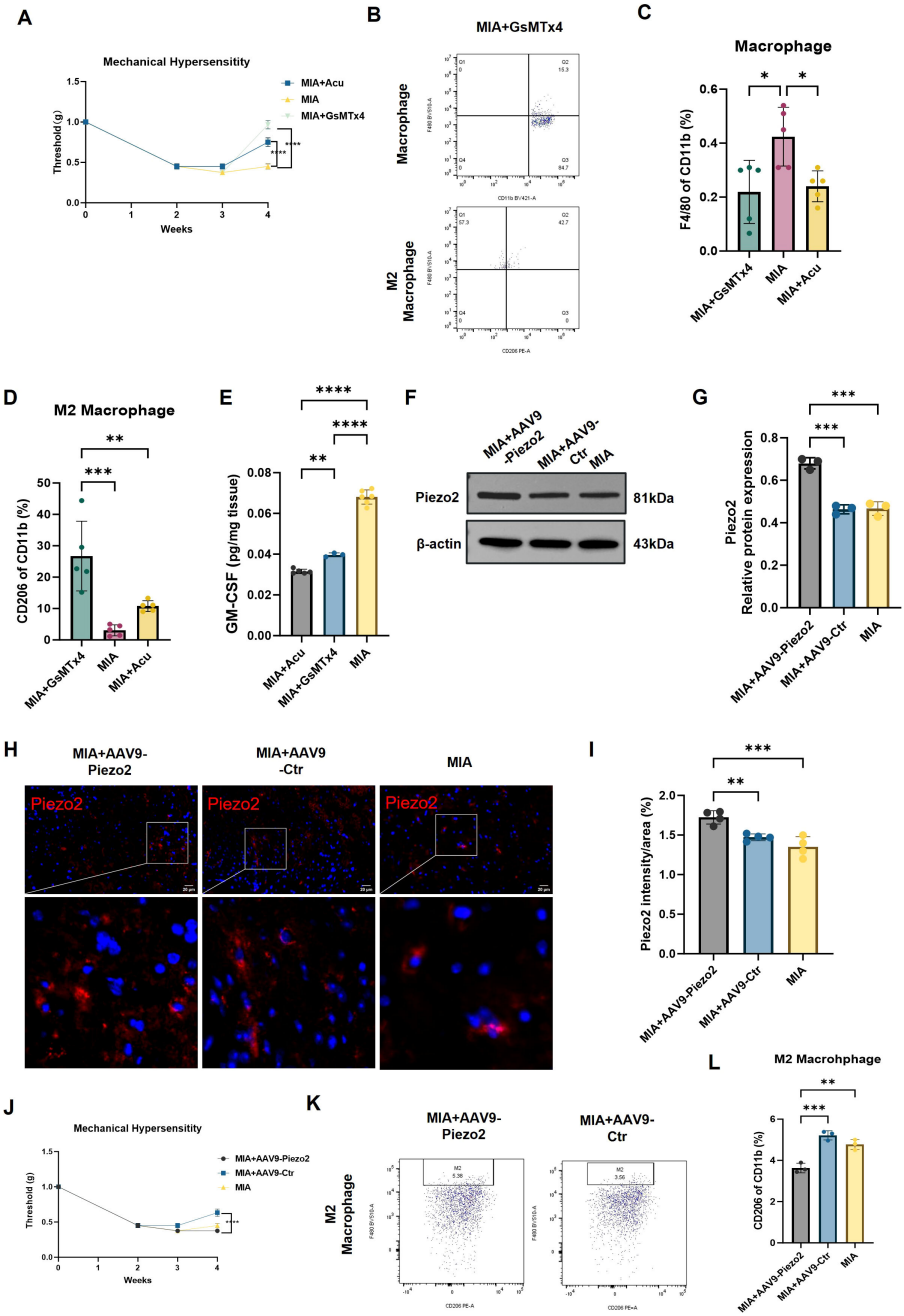
Piezo2 inhibition attenuates OA pain and remodels the DRG immune microenvironment. **(A)** Time-course changes in mechanical withdrawal thresholds measured by von Frey test in MIA, MIA+Acupuncture (Acu), and MIA+GsMTx4 (Piezo channel inhibitor) groups. **(B)** Representative flow cytometry plots showing gating strategy for macrophages (F4/80^+^CD11b^+^) and M2 macrophages (F4/80^+^CD206^+^) in L3-L5 dorsal root ganglia (DRGs) from MIA+GsMTx4 mice. **(C, D)** Quantitative analysis of the percentage of F4/80^+^CD11b^+^ macrophages **(C)** and F4/80^+^CD206^+^ M2 macrophages **(D)** in DRGs. **(E)** ELISA quantification of GM-CSF levels in DRG tissue lysates from MIA, MIA+GsMTx4, and MIA+Acu groups. **(F)** Western blot analysis of Piezo2 protein expression in DRGs from MIA, MIA+AAV9-Ctr (control virus), and MIA+AAV9-Piezo2 (Piezo2 activation) groups. β-actin was used as a loading control. **(G)** Densitometric quantification of Piezo2 protein levels normalized to β-actin. **(H)** Representative immunofluorescence images of Piezo2 (red) in DRG sections from MIA+AAV9-Piezo2, MIA+AAV9-Ctr, and MIA groups. Nuclei were counterstained with DAPI (blue). Scale bars represent 50 μm (upper panel) and 20 μm (lower panel). **(I)** Quantitative analysis of Piezo2 fluorescence intensity in DRG sections. **(J)** Time-course evaluation of mechanical withdrawal thresholds in MIA, MIA+AAV9-Ctr, and MIA+AAV9-Piezo2 groups. **(K)** Representative flow cytometry plots showing M2 macrophage (F4/80^+^CD206^+^) populations in DRGs from MIA+AAV9-Piezo2 and MIA+AAV9-Ctr mice. **(L)** Quantitative analysis of the percentage of F4/80^+^CD206^+^ M2 macrophages in DRGs. Data are presented as mean ± SEM (n = 5–6 per group). Statistical significance was determined by two-way repeated-measures ANOVA followed by Bonferroni *post-hoc* test for **(A, J)**, and one-way ANOVA followed by Tukey’s *post-hoc* test for **(C, D, E, G, I, L)**. *P < 0.05, **P < 0.01, ***P < 0.001, ****P < 0.0001 vs. MIA group; ****P < 0.0001 vs. MIA+Acu group **(A)**. The schematic in panel A was created using FigDraw.

To determine whether Piezo2 acts upstream of macrophage polarization and GM-CSF signaling, MIA mice were treated with the Piezo channel inhibitor GsMTx4. Densitometric analysis showed that Piezo1 protein was weakly detected in DRG tissues and did not differ significantly among the indicated groups (**Supplementary Figures 3E, F**). Pharmacological inhibition of Piezo channels alleviated mechanical allodynia, reduced total macrophage abundance, increased M2 macrophage proportion, and decreased GM-CSF levels in DRGs (**Figures 6A–E**; **Supplementary Figures 3G, H**). These effects closely mimicked acupuncture treatment.

Conversely, neuron-specific overactivation of Piezo2 in DRGs was achieved using AAV9-hSyn-Piezo2. Western blot and immunofluorescence confirmed successful overexpression of Piezo2 (**Figures 6F–I**). Piezo2 activation exacerbated mechanical allodynia (**Figure 6J**) and induced pro-inflammatory immune remodeling: increased CD45^+^ immune cell infiltration, elevated macrophage ratio, and reduced M2 macrophage proportion (**Figures 6K, L**). Together, the pharmacological inhibition and neuron-specific Piezo2 overexpression experiments support an important role for Piezo2-associated signaling in DRG immune remodeling and mechanical hypersensitivity.

### 3.7 Inhibition of Piezo2 in DRG neurons promotes a CD206-positive macrophage phenotype *in vitro*

**Figure 7:**
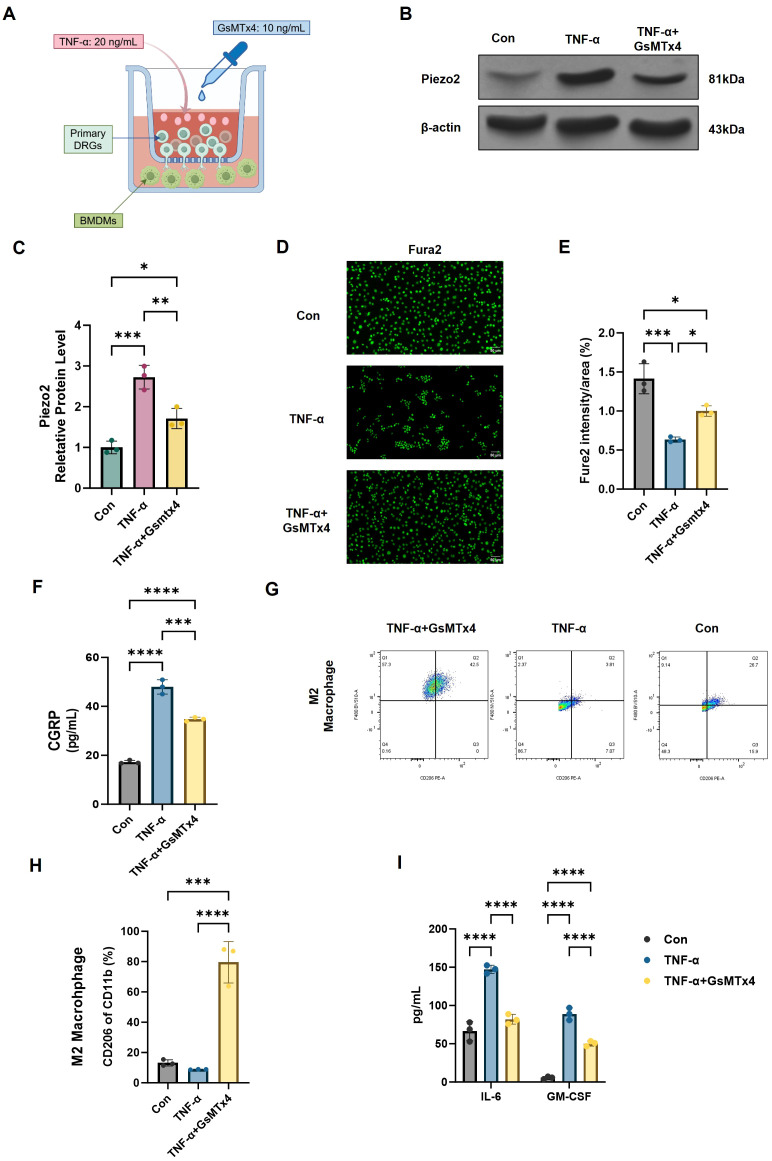
Inhibition of Piezo2 in DRG neurons promotes a CD206-positive macrophage phenotype *in vitro.* **(A)** Schematic illustration of the *in vitro* conditioned medium (CM) experimental design. Primary dorsal root ganglion (DRG) neurons were pre-stimulated with TNF-α (20 ng/mL) to mimic an inflammatory microenvironment, followed by treatment with the Piezo channel inhibitor GsMTx4 (10 ng/mL). The resulting neuronal CM was collected and applied to bone marrow-derived macrophages (BMDMs) for functional assays. **(B)** Representative Western blot images showing Piezo2 protein expression in primary DRG neurons across the Control (Con), TNF-α, and TNF-α+GsMTx4 groups. β-actin was used as the internal loading control. **(C)** Densitometric quantification of Piezo2 protein levels, normalized to β-actin. **(D)** Representative Fura-2 calcium imaging fluorescence micrographs of primary DRG neurons in each experimental group. Scale bar: 50 μm. **(E)** Quantitative analysis of Fura-2 calcium fluorescence signals in primary DRG neurons. **(F)** ELISA quantification of calcitonin gene-related peptide (CGRP) concentrations in the neuronal CM. **(G)** Representative flow cytometry dot plots demonstrating the gating strategy for M2 macrophages (F4/80^+^CD206^+^) in BMDMs cultured with CM from Con, TNF-α, and TNF-α+GsMTx4-treated DRG neurons. **(H)** Quantitative analysis of the percentage of F4/80^+^CD206^+^ M2 macrophages in BMDMs. **(I)** ELISA quantification of IL-6 and GM-CSF levels in BMDM culture supernatants. Data are presented as mean ± standard error of the mean (SEM) from n = 3–5 independent biological replicates. Statistical analyses were performed using one-way ANOVA followed by Tukey’s multiple comparisons *post-hoc* test. *P < 0.05, **P < 0.01, ***P < 0.001, ****P < 0.0001, as indicated. The schematic in panel A was created using FigDraw.

To further examine the interaction between neuronal Piezo2 activity and macrophage phenotype, we performed conditioned medium (CM) experiments using primary DRG neurons and bone marrow–derived macrophages (BMDMs) (**Figure 7A**). DRG neurons were pretreated with TNF−α to mimic an inflammatory microenvironment, followed by GsMTx4.

GsMTx4 treatment reduced Piezo2 expression in DRG neurons (**Figures 7B, C**). Representative Fura-2 calcium imaging and quantitative fluorescence analysis showed that TNF-α stimulation increased neuronal Ca²^+^ signals, whereas GsMTx4 treatment attenuated this increase (**Figures 7D, E**). Consistently, GsMTx4 decreased CGRP release in neuronal conditioned medium (**Figure 7F**). BMDMs incubated with CM from GsMTx4-treated neurons showed a significantly higher proportion of F4/80^+^CD206^+^ CD206-positive/M2-like macrophages (**Figures 7G, H**) and reduced secretion of IL-6 and GM-CSF (**Figure 7I**). These *in vitro* data suggest that suppressing Piezo2 activity in sensory neurons can shift macrophages toward a CD206-positive phenotype via paracrine signaling.

## 4 Discussion

Osteoarthritis (OA) is a leading cause of chronic musculoskeletal pain characterized by prominent mechanical allodynia and persistent low−grade inflammation. Accumulating evidence indicates that neuro−immune crosstalk in the dorsal root ganglion (DRG) acts as a central driver of peripheral sensitization and pain persistence. Acupuncture at ST36 has been widely recognized for its analgesic efficacy in OA (30, 31), yet the molecular link between its mechanical stimulus and DRG immune remodeling remains poorly understood. In this study, we demonstrate that manual acupuncture alleviates OA pain by reshaping the DRG neuro−immune microenvironment through a Piezo2−GM−CSF−macrophage polarization axis. Our findings establish Piezo2 as a critical mechanotransducer that bridges peripheral mechanical stimulation to neuronal−immune communication, providing a novel mechanistic basis for acupuncture analgesia.

Chronic OA pain is no longer regarded as a mere consequence of structural joint damage, but rather as a neuro−immune disorder driven by aberrant sensory signaling and dysregulated inflammation. The DRG serves as a pivotal hub where primary sensory neurons and resident immune cells dynamically interact to modulate nociceptive excitability. Inflammatory mediators released within the DRG promote macrophage infiltration, pro−inflammatory polarization, and sustained nociceptor sensitization. Consistent with previous reports, our study confirmed that MIA−induced OA triggers robust neuroinflammation in the DRG, accompanied by mechanical hypersensitivity. Notably, we found that acupuncture exerted negligible effects on systemic cytokines but strongly suppressed inflammatory profiles specifically within the DRG. This regional immunomodulation highlights the DRG as a primary anatomical target underlying acupuncture−mediated pain relief, rather than generalized anti−inflammatory actions.

Macrophages are the most abundant immune cells in the DRG and play a dual role in pain regulation. Pro−inflammatory M1 macrophages exacerbate neuroinflammation and pain, whereas anti−inflammatory M2 macrophages facilitate inflammation resolution and tissue repair (32). Our flow cytometric and immunofluorescent results revealed that acupuncture markedly reduced total macrophage abundance while skewing macrophage polarization toward an M2 phenotype in the DRG. These observations align with previous studies showing that acupuncture can modulate macrophage plasticity in inflammatory and neuropathic pain models (33). By promoting a reparative macrophage phenotype, acupuncture may interrupt the self−reinforcing cycle of neuronal hyperexcitability and inflammation, thereby alleviating mechanical allodynia. Our transcriptomic analysis further supported this notion by identifying enrichment of pathways related to efferocytosis, PI3K−Akt, and MAPK signaling, all of which are closely associated with macrophage polarization and immune resolution.

A key finding of this study is the identification of Piezo2 as a master regulator linking mechanical acupuncture signals to DRG neuro−immune remodeling. Piezo2 is a well−characterized mechanosensitive ion channel highly expressed in DRG nociceptors and essential for mechanical pain sensation (13, 34). Previous studies demonstrated that Piezo2 is upregulated in OA and contributes to injury−induced mechanical allodynia (12). In line with these reports, we found that Piezo2 expression was increased in knee-innervating DRG neurons in MIA-induced OA mice and was reduced after acupuncture treatment. Importantly, acupuncture substantially reduced Piezo2 expression and function in these neurons. Because acupuncture is inherently a controlled mechanical stimulus, we propose that repetitive, mild mechanical stimulation at ST36 normalizes pathologically enhanced Piezo2 activity, thereby breaking the vicious cycle of mechanotransduction−driven neuroinflammation.

We further identified GM-CSF as a pain-related inflammatory mediator associated with Piezo2-dependent remodeling of the DRG microenvironment. GM-CSF is a cytokine involved in myeloid-cell activation, and its role in inflammatory and arthritic pain has been recognized. Our Luminex, ELISA, and immunofluorescence data showed that GM-CSF was low under basal conditions, increased in MIA-induced DRGs, and was reduced after acupuncture. Neutralization of GM-CSF significantly ameliorated OA pain, indicating that GM-CSF contributes to the maintenance of mechanical hypersensitivity. Conversely, overactivation of Piezo2 in knee-innervating DRG sensory neuron increased GM−CSF production, promoted pro−inflammatory macrophage profiles, and exacerbated pain hypersensitivity (27–29). However, because macrophage phenotypes after GM-CSF neutralization were not directly examined, we interpret GM-CSF as a pain-related inflammatory mediator rather than as a direct mediator of macrophage polarization.

Our *in vitro* conditioned medium experiments provided direct mechanistic evidence that Piezo2 inhibition in DRG neurons is sufficient to drive macrophages toward an anti−inflammatory phenotype. TNF−α−activated DRG neurons released pro−inflammatory factors that maintained macrophages in a pro−inflammatory state; in contrast, inhibiting Piezo2 shifted the neuronal secretome toward an anti−inflammatory profile characterized by reduced CGRP, IL−6, and GM−CSF. These results confirm that Piezo2−dependent paracrine signaling from sensory neurons directly dictates macrophage fate, reinforcing the concept of DRG neurons as active modulators of local immunity rather than only passive signal conductors (35, 36).

Several limitations should be acknowledged. First, GsMTx4 is not strictly specific to Piezo2 and may also inhibit Piezo1. Although our data suggest Piezo2 is the dominant player in this context, future studies using Piezo2 conditional knockout mice will be required to confirm its cell−specific role. Second, the revised images demonstrate Piezo2 expression in FG-labeled knee-innervating DRG neurons and partial overlap with CGRP-positive neurons, but additional co-staining with IB4 and NF200 would be needed to fully define the distribution of Piezo2 across non-peptidergic and myelinated DRG neuronal subtypes. Third, although our data show acupuncture-associated reductions in total macrophage accumulation and increases in the CD206-positive/M2-like macrophage phenotype, M1-like macrophages were not systematically assessed by flow cytometry in this study. Additional analyses using M1-associated markers such as CD86, MHC-II, or iNOS will be needed to determine whether acupuncture also modulates M1-like macrophage infiltration in the DRG. Fourth, the precise transcriptional or post-translational mechanisms by which acupuncture downregulates Piezo2 remain unclear and warrant further investigation. Finally, only male mice were used in this study. Given known sex differences in pain processing, DRG immune responses, macrophage activation, and mechanotransduction pathways, whether this Piezo2-associated neuro-immune mechanism and the analgesic effect of acupuncture are conserved in female mice requires future investigation.

In conclusion, our study reveals a neuro−immune mechanism underlying acupuncture analgesia in OA. Mechanical stimulation at ST36 suppresses Piezo2 in DRG nociceptors, reduces GM−CSF secretion, and redirects macrophage polarization toward an anti−inflammatory M2 phenotype, thereby remodeling the DRG neuro−immune microenvironment and alleviating mechanical allodynia. These findings position Piezo2−associated neuro−immune crosstalk as a promising therapeutic target for OA pain and provide a mechanistic framework to optimize acupuncture−based therapies.

## 5 Conclusion

In conclusion, our study demonstrates that manual acupuncture at ST36 alleviates osteoarthritis pain by remodeling the DRG neuro-immune microenvironment. Acupuncture downregulates Piezo2 in knee-innervating DRG sensory neurons, reduces inflammatory mediators including GM-CSF, and promotes a CD206-positive/M2-like macrophage phenotype. These findings identify Piezo2 as a key mechanotransducer and potential therapeutic target for OA pain, providing new insights into neuro-immune crosstalk modulation by peripheral mechanical stimulation.

## Data Availability

The datasets presented in this study can be found in online repositories. The names of the repository/repositories and accession number(s) can be found below: GSE327205 (GEO).
