# Supplementary material for: Acupuncture alleviates osteoarthritis pain by remodeling the DRG neuro-immune microenvironment via Piezo2-mediated macrophage polarization
Source: Front Immunol. 2026 Jul 30;17:1844125. doi: 10.3389/fimmu.2026.1844125 (PMC13471090; doi:10.3389/fimmu.2026.1844125)
Supplement: Supplementary file 1 [file DataSheet2.pdf]

frontiers

The International Society for the Advancement of Cytometry (ISAC) has highlighted the importance of including comprehensive methodological information to ensure data reproducibility and reliability. In line with this, Frontiers in Immunology now requires authors to submit a checklist for manuscripts that involve flow or mass cytometry. This checklist helps standardize the reporting process, improving the quality and transparency of published data. By doing so, we support scientific progress, making it easier for other researchers to replicate and validate experiments.

This form should be submitted with any manuscripts using flow or mass cytometry.

Sample/specimen/material description

☐ Total blood

☐ PBMCs

☑ Organ digests

Other Mouse dorsal root ganglia (DRG)

Did the samples suffer any treatment before or after incubation with the antibodies?

☐ Drug ______________________________

☑ Cell permeabilization ____________________

☐ Dye ______________________________

☐ Propidium iodine

☐ Not applicable

Other TruStain FcXTM blocking

Instrument and antibodies

Name of the Cytometer BD LSRFortessa

Antibodies and targets	Fluorochrome/ Metal	Catalog number/Company
e.g. anti-CD4	FITC	Cat. XXX/ XXX ltd.
PerCP/Cyanine5.5 anti-mouse CD45	PerCP/Cy5.5	103132 / BioLegend

anti-mouse/human CD11b	BV421	101235 / BioLegend
FITC anti-mouse Ly-6G	FITC	127605 / BioLegend
APC/Cyanine7 anti-mouse Ly-6G/Ly-6C (Gr-1)	APC/Cy7	108423 / BioLegend
PerCP/Fire? 806 anti-mouse F4/80	PerCP/Fire806	123181 / BioLegend
PE anti-mouse CD206 (MMR)	PE	141705 / BioLegend
Brilliant Violet 605 anti-mouse CD3	BV605	100237 / BioLegend
APC anti-mouse/human CD45R/B220	APC	103212 / BioLegend
TruStain FcX? (anti-mouse CD16/32)		101319 / BioLegend

Data analyses

1. Name of the software FlowJo Version 10.8.1__________________________________
2. Reference gating strategy in the manuscript or supplementary material

Gating strategy in (eg Figure X) Figure S1D, Figure 3A, Figure 6B, Figure 6K, Figure 7F ______________________________
