# Supplementary material for: Acupuncture alleviates osteoarthritis pain by remodeling the DRG neuro-immune microenvironment via Piezo2-mediated macrophage polarization
Source: Front Immunol. 2026 Jul 30;17:1844125. doi: 10.3389/fimmu.2026.1844125 (PMC13471090; doi:10.3389/fimmu.2026.1844125)
Supplement: Supplementary file 2 [file DataSheet1.docx]

Supplementary Material

# 1 Supplementary Figures and Tables

## 1.1 Supplementary Figures

## Supplementary Figure 1

**Figure S1. Supplementary validation of MIA-induced OA pathology, DRG neuronal tracing, and immune cell infiltration**

(A) Representative Safranin O-fast green staining images of knee joint sections from MIA-injected and Control (Con) mice, demonstrating extensive articular cartilage erosion in MIA animals. Red boxes indicate the region of articular cartilage evaluated for OARSI scoring. Scale bar: 50 μm. (B) Quantitative analysis of the OARSI score confirming severe cartilage damage in the MIA group compared to the Con group. (C) Representative Fluoro-Gold (FG) retrograde labeling images in L2, L4, and S1 dorsal root ganglia (DRGs), showing that the knee joint is primarily innervated by L4 DRG neurons. Scale bar: 50 μm. (D) Representative flow cytometry gating strategy for immune cell subpopulations, including lymphocytes, macrophages, neutrophils, and monocytes, isolated from L3-L5 DRGs. (E,F) Quantitative analysis of the percentage of Ly6G⁺ neutrophils (E) and Ly6C⁺ monocytes (F) within the CD11b⁺ myeloid cell population in DRGs from MIA+Acupuncture (Acu) and MIA groups. Data are presented as mean ± SEM (n = 5–6 per group). Statistical significance was determined by unpaired Student’s t-test. *P < 0.05, **P < 0.01, ***P < 0.001, as indicated.

## Supplementary Figure 2

**Figure S2. Baseline macrophage marker staining and exploratory assessment of M1-like macrophage markers in DRG sections.**

(A) Representative immunofluorescence image showing F4/80 (red), CD206 (green), and DAPI (blue) staining in ipsilateral L3–L5 DRG sections from the Control (Con) group. This image was used as a baseline reference for macrophage and CD206-positive/M2-like macrophage signals under normal conditions. (B) Representative immunofluorescence images showing F4/80 (red), iNOS (green), and DAPI (blue) staining in ipsilateral L3–L5 DRG sections from the Con, MIA, and MIA + Acu groups. F4/80 was used as a pan-macrophage marker, and iNOS was used as an M1-associated marker. Under the present staining conditions, iNOS-positive F4/80-positive macrophages were barely detectable in DRG sections; therefore, these images are presented as exploratory staining results, and no definitive quantitative conclusion regarding M1-like macrophage changes was drawn. Scale bars, 20 μm.

## Supplementary Figure 3

**Figure S3. GM-CSF neutralization, macrophage depletion, and Piezo channel expression in DRGs**

(A) ELISA quantification of granulocyte-macrophage colony-stimulating factor (GM-CSF) levels in ipsilateral L3–L5 DRG tissue lysates from the Con, MIA, MIA + Acu, and MIA + anti-GM-CSF neutralizing antibody groups. (B) Representative immunofluorescence image showing GM-CSF, F4/80, CD206, and DAPI staining in Control DRG sections. GM-CSF signals were weak under basal conditions in Con DRGs. Scale bar, 20 μm. (C) Representative flow-cytometry plots showing F4/80⁺CD11b⁺ macrophage populations in DRGs from the MIA and MIA + clodronate liposome (CL, macrophage depletion) groups. (D) Quantitative analysis of the percentage of F4/80⁺CD11b⁺ macrophages in DRGs after CL-mediated macrophage depletion. (E) Western blot analysis of Piezo1 protein expression in L3–L5 DRGs from the Con, MIA, and MIA + Acu groups. β-actin was used as the loading control. (F) Densitometric quantification of Piezo1 protein levels normalized to β-actin. (G) Western blot analysis of Piezo2 protein expression in L3–L5 DRGs from the MIA, MIA + Acu, and MIA + GsMTx4 groups. β-actin was used as the loading control. (H) Densitometric quantification of Piezo2 protein levels normalized to β-actin. Data are presented as mean ± SEM. Statistical significance was determined by unpaired Student’s t-test for two-group comparisons and one-way ANOVA followed by Tukey’s post hoc test for multiple-group comparisons. *P < 0.05, **P < 0.01, ***P < 0.001, ****P < 0.0001.

## 1.2 Supplementary Tables

## Supplementary Table 1

| Differentially expressed genes in the transcriptome | | | | |
| --- | --- | --- | --- | --- |
| gene_name | gene_id | log2FoldChange | pvalue | padj |
| Tinagl1 | ENSMUSG00000028776 | 0.827644475 | 9.90E-13 | 1.32E-08 |
| Tbx1 | ENSMUSG00000009097 | 1.886715619 | 1.77E-12 | 1.32E-08 |
| Pappa2 | ENSMUSG00000073530 | -1.030102692 | 2.78E-12 | 1.38E-08 |
| Cldn5 | ENSMUSG00000041378 | 0.736132808 | 2.38E-10 | 8.87E-07 |
| Ly6c1 | ENSMUSG00000079018 | 0.685788355 | 3.28E-10 | 9.78E-07 |
| Piezo2 | ENSMUSG00000041482 | -0.516721494 | 1.93E-09 | 4.79E-06 |
| Prune2 | ENSMUSG00000039126 | -0.537794632 | 3.86E-09 | 8.21E-06 |
| Rtl9 | ENSMUSG00000085584 | -1.173435212 | 1.71E-08 | 2.83E-05 |
| Mrgprb5 | ENSMUSG00000070551 | -0.606861375 | 2.01E-08 | 2.99E-05 |
| Asic3 | ENSMUSG00000038276 | -0.595190095 | 2.51E-08 | 3.40E-05 |
| Klhl23 | ENSMUSG00000042155 | 0.664679799 | 2.87E-08 | 3.57E-05 |
| Ahnak2 | ENSMUSG00000072812 | -0.515116177 | 4.40E-08 | 5.04E-05 |
| C1qtnf6 | ENSMUSG00000022440 | 1.445044063 | 5.49E-08 | 5.81E-05 |
| Erich3 | ENSMUSG00000078161 | -0.53601237 | 5.84E-08 | 5.81E-05 |
| Dock3 | ENSMUSG00000039716 | -0.515153764 | 1.01E-07 | 8.72E-05 |
| Defb42 | ENSMUSG00000054763 | -0.705905047 | 1.21E-07 | 9.46E-05 |
| Map1b | ENSMUSG00000052727 | -0.511354665 | 1.35E-07 | 0.000100855 |
| Slco1c1 | ENSMUSG00000030235 | 0.990315869 | 1.56E-07 | 0.000110859 |
| Afap1l1 | ENSMUSG00000033032 | 0.736387982 | 1.74E-07 | 0.000117819 |
| Susd2 | ENSMUSG00000006342 | -0.53526961 | 2.87E-07 | 0.000169737 |
| Atp8a2 | ENSMUSG00000021983 | -0.526624061 | 3.22E-07 | 0.000171373 |
| mt-Rnr1 | ENSMUSG00000064337 | 0.756512138 | 5.04E-07 | 0.000250622 |
| Cyr61 | ENSMUSG00000028195 | 1.104334594 | 5.37E-07 | 0.00025822 |
| Magix | ENSMUSG00000031147 | 1.021516668 | 6.37E-07 | 0.000277695 |
| Cdhr1 | ENSMUSG00000021803 | -0.577569073 | 6.37E-07 | 0.000277695 |
| Serpinh1 | ENSMUSG00000070436 | 1.069574053 | 6.61E-07 | 0.000277695 |
| Mettl7a3 | ENSMUSG00000058057 | -0.578106081 | 6.70E-07 | 0.000277695 |
| Wisp1 | ENSMUSG00000005124 | 0.945727226 | 7.06E-07 | 0.000284743 |
| Oprd1 | ENSMUSG00000050511 | -0.617628301 | 8.50E-07 | 0.000325153 |
| mt-Rnr2 | ENSMUSG00000064339 | 0.809847156 | 1.08E-06 | 0.000383336 |
| Ntrk3 | ENSMUSG00000059146 | -0.604697828 | 1.30E-06 | 0.000383336 |
| Ptprb | ENSMUSG00000020154 | 0.744243046 | 1.32E-06 | 0.000383336 |
| Plekhd1 | ENSMUSG00000066438 | -0.505783598 | 1.38E-06 | 0.000383336 |
| Ryr2 | ENSMUSG00000021313 | -0.679149819 | 1.39E-06 | 0.000383336 |
| Map1a | ENSMUSG00000027254 | -0.523841022 | 1.57E-06 | 0.000410513 |
| Necap2 | ENSMUSG00000028923 | 0.54003635 | 1.62E-06 | 0.000417156 |
| Alad | ENSMUSG00000028393 | 0.529557852 | 1.75E-06 | 0.000432129 |
| Emid1 | ENSMUSG00000034164 | 0.937401163 | 1.80E-06 | 0.000432129 |
| Timp4 | ENSMUSG00000030317 | 1.517938747 | 2.24E-06 | 0.0005143 |
| Apln | ENSMUSG00000037010 | 0.959079247 | 2.35E-06 | 0.000528591 |
| Myl9 | ENSMUSG00000067818 | 0.825644461 | 2.44E-06 | 0.000528591 |
| Gpx8 | ENSMUSG00000021760 | 0.788604706 | 2.45E-06 | 0.000528591 |
| Tcf7l2 | ENSMUSG00000024985 | 0.727108431 | 2.78E-06 | 0.000581411 |
| Apcdd1 | ENSMUSG00000071847 | 0.567649321 | 2.81E-06 | 0.000581411 |
| Epha2 | ENSMUSG00000006445 | 0.847147314 | 2.94E-06 | 0.000597458 |
| Tnfrsf19 | ENSMUSG00000060548 | 0.817724331 | 3.16E-06 | 0.000608875 |
| Sorl1 | ENSMUSG00000049313 | -0.501963873 | 3.18E-06 | 0.000608875 |
| Adgrl4 | ENSMUSG00000039167 | 0.609131646 | 3.33E-06 | 0.000628832 |
| Pltp | ENSMUSG00000017754 | 0.586346824 | 3.72E-06 | 0.00069263 |
| Sprr1a | ENSMUSG00000050359 | -5.622124542 | 5.15E-06 | 0.000909742 |
| Jdp2 | ENSMUSG00000034271 | 0.527065389 | 5.20E-06 | 0.000909742 |
| Kdelr3 | ENSMUSG00000010830 | 0.996554986 | 5.94E-06 | 0.000982013 |
| Oprm1 | ENSMUSG00000000766 | -0.60649233 | 6.03E-06 | 0.000982013 |
| Col4a5 | ENSMUSG00000031274 | 1.165394918 | 6.03E-06 | 0.000982013 |
| Gm14025 | ENSMUSG00000079051 | -0.63393985 | 6.06E-06 | 0.000982013 |
| P3h1 | ENSMUSG00000028641 | 0.756225934 | 6.31E-06 | 0.000995508 |
| Inpp5j | ENSMUSG00000034570 | -0.512500759 | 6.42E-06 | 0.000995873 |
| Plxna2 | ENSMUSG00000026640 | -0.561013892 | 6.48E-06 | 0.000995873 |
| Adam11 | ENSMUSG00000020926 | -0.507377697 | 6.70E-06 | 0.001004696 |
| Tmem38b | ENSMUSG00000028420 | 0.620676866 | 6.74E-06 | 0.001004696 |
| Nts | ENSMUSG00000019890 | -1.173417813 | 7.21E-06 | 0.001038911 |
| Nedd1 | ENSMUSG00000019988 | 0.635977341 | 8.13E-06 | 0.001092859 |
| Cyth4 | ENSMUSG00000018008 | 0.544779746 | 8.29E-06 | 0.001101291 |
| Ltbr | ENSMUSG00000030339 | 0.594837584 | 8.85E-06 | 0.001118104 |
| Gata2 | ENSMUSG00000015053 | 0.85474682 | 9.57E-06 | 0.00119894 |
| Inpp4b | ENSMUSG00000037940 | 0.977167005 | 9.79E-06 | 0.001203949 |
| Mylip | ENSMUSG00000038175 | 0.530263329 | 9.85E-06 | 0.001203949 |
| Adra2a | ENSMUSG00000033717 | 0.940493315 | 1.08E-05 | 0.00130464 |
| Lamc3 | ENSMUSG00000026840 | 0.673199895 | 1.19E-05 | 0.001408457 |
| Robo4 | ENSMUSG00000032125 | 0.558856512 | 1.20E-05 | 0.001414626 |
| Dgki | ENSMUSG00000038665 | -0.620957655 | 1.23E-05 | 0.00143398 |
| Tsku | ENSMUSG00000049580 | 1.12704931 | 1.24E-05 | 0.001438698 |
| Egln1 | ENSMUSG00000031987 | 0.557992993 | 1.28E-05 | 0.001465657 |
| Gpx7 | ENSMUSG00000028597 | 0.79852295 | 1.29E-05 | 0.001472782 |
| Tesk2 | ENSMUSG00000033985 | 0.872423148 | 1.31E-05 | 0.001475541 |
| Plxna4 | ENSMUSG00000029765 | -0.535280787 | 1.52E-05 | 0.001610341 |
| Srrm4 | ENSMUSG00000063919 | -0.510627664 | 1.57E-05 | 0.001642302 |
| Cd38 | ENSMUSG00000029084 | 0.816828879 | 1.68E-05 | 0.001703698 |
| Tmem267 | ENSMUSG00000074634 | -0.758760954 | 1.70E-05 | 0.001703698 |
| Ksr2 | ENSMUSG00000061578 | -0.548916783 | 1.89E-05 | 0.001870297 |
| Dusp18 | ENSMUSG00000047205 | 0.634792126 | 1.93E-05 | 0.001889026 |
| Col26a1 | ENSMUSG00000004415 | 1.181897981 | 1.94E-05 | 0.001894343 |
| Grm8 | ENSMUSG00000024211 | -0.597389546 | 1.99E-05 | 0.001917232 |
| Plod1 | ENSMUSG00000019055 | 0.628335702 | 2.14E-05 | 0.00202098 |
| Mdga1 | ENSMUSG00000043557 | -0.582279097 | 2.22E-05 | 0.002052518 |
| Tfpi | ENSMUSG00000027082 | 0.733163714 | 2.23E-05 | 0.002052518 |
| Rasgrf2 | ENSMUSG00000021708 | -0.512017312 | 2.33E-05 | 0.002082108 |
| Pacsin3 | ENSMUSG00000027257 | 0.796004977 | 2.39E-05 | 0.002112613 |
| Cd83 | ENSMUSG00000015396 | 0.784763698 | 2.60E-05 | 0.002225063 |
| Dap | ENSMUSG00000039168 | 0.565449361 | 2.64E-05 | 0.002235505 |
| Zfp651 | ENSMUSG00000013419 | 0.565873291 | 2.64E-05 | 0.002235505 |
| Fam57a | ENSMUSG00000069808 | 0.956752615 | 2.65E-05 | 0.002235505 |
| Calcoco2 | ENSMUSG00000006056 | 1.691082059 | 3.00E-05 | 0.00244576 |
| Sox11 | ENSMUSG00000063632 | -0.526546095 | 3.05E-05 | 0.002457405 |
| Cntnap4 | ENSMUSG00000031772 | -0.626447132 | 3.08E-05 | 0.002457405 |
| Ucp2 | ENSMUSG00000033685 | 0.632355678 | 3.83E-05 | 0.002788188 |
| Col5a2 | ENSMUSG00000026042 | 0.815032007 | 4.02E-05 | 0.002912258 |
| Crmp1 | ENSMUSG00000029121 | -0.509860548 | 4.09E-05 | 0.002947893 |
| Tmem204 | ENSMUSG00000024168 | 0.632759257 | 4.38E-05 | 0.003083123 |
| Sox7 | ENSMUSG00000063060 | 0.951730641 | 4.49E-05 | 0.003110412 |
| Adam17 | ENSMUSG00000052593 | 0.556510124 | 4.51E-05 | 0.003110412 |
| Tmem150a | ENSMUSG00000055912 | 0.769263035 | 4.55E-05 | 0.003110412 |
| Celsr3 | ENSMUSG00000023473 | -0.507284144 | 4.59E-05 | 0.003123858 |
| Arhgap36 | ENSMUSG00000036198 | -0.519666763 | 4.83E-05 | 0.003248378 |
| Myl12a | ENSMUSG00000024048 | 0.630415786 | 4.86E-05 | 0.003248378 |
| Sparc | ENSMUSG00000018593 | 0.908998314 | 5.04E-05 | 0.003355843 |
| Edn1 | ENSMUSG00000021367 | 0.91947221 | 5.29E-05 | 0.003407722 |
| Ppp2r5a | ENSMUSG00000026626 | 0.58432922 | 5.29E-05 | 0.003407722 |
| Lrtm2 | ENSMUSG00000055003 | 1.292305214 | 5.31E-05 | 0.003407722 |
| Bmf | ENSMUSG00000040093 | 0.500474647 | 5.32E-05 | 0.003407722 |
| Ppic | ENSMUSG00000024538 | 1.28865115 | 5.49E-05 | 0.003409859 |
| Srgn | ENSMUSG00000020077 | 0.557998864 | 5.61E-05 | 0.003458647 |
| Pi15 | ENSMUSG00000067780 | 0.788229881 | 5.82E-05 | 0.003532242 |
| N4bp3 | ENSMUSG00000001053 | 0.764174996 | 5.87E-05 | 0.003546448 |
| Ryk | ENSMUSG00000032547 | 0.681017972 | 6.01E-05 | 0.003600904 |
| Cd93 | ENSMUSG00000027435 | 0.628741872 | 6.02E-05 | 0.003600904 |
| Rnf128 | ENSMUSG00000031438 | 0.634597424 | 6.04E-05 | 0.003600904 |
| Chst1 | ENSMUSG00000027221 | 0.530108092 | 6.34E-05 | 0.003709254 |
| Papss2 | ENSMUSG00000024899 | 1.155085067 | 6.34E-05 | 0.003709254 |
| Pex2 | ENSMUSG00000040374 | 0.509405154 | 6.43E-05 | 0.003743949 |
| Tmem134 | ENSMUSG00000024845 | 0.624282811 | 6.45E-05 | 0.003743949 |
| Rbp1 | ENSMUSG00000046402 | 0.742309713 | 6.52E-05 | 0.003766436 |
| Col9a3 | ENSMUSG00000027570 | 1.496858687 | 6.75E-05 | 0.003814411 |
| Pde7b | ENSMUSG00000019990 | 0.545787206 | 6.92E-05 | 0.003872096 |
| Insc | ENSMUSG00000048782 | 0.5414205 | 6.93E-05 | 0.003872096 |
| Kcnk18 | ENSMUSG00000040901 | -0.600217156 | 6.97E-05 | 0.003879467 |
| Col5a1 | ENSMUSG00000026837 | 0.958307704 | 7.07E-05 | 0.00392214 |
| Ccdc172 | ENSMUSG00000025090 | -0.668259428 | 7.19E-05 | 0.003958652 |
| Eng | ENSMUSG00000026814 | 0.609016384 | 7.41E-05 | 0.004019844 |
| Pcdhac1 | ENSMUSG00000103255 | -0.52687888 | 7.82E-05 | 0.004163381 |
| Rhoj | ENSMUSG00000046768 | 0.773571352 | 8.08E-05 | 0.004247535 |
| Fam198b | ENSMUSG00000027955 | 0.631880666 | 8.81E-05 | 0.004543965 |
| Aff2 | ENSMUSG00000031189 | -0.662741919 | 8.85E-05 | 0.004543965 |
| Ajuba | ENSMUSG00000022178 | 0.80341264 | 8.87E-05 | 0.004543965 |
| Tie1 | ENSMUSG00000033191 | 0.533917496 | 9.00E-05 | 0.004561603 |
| Dll1 | ENSMUSG00000014773 | 1.170952107 | 9.02E-05 | 0.004561603 |
| Samd9l | ENSMUSG00000047735 | 0.582843186 | 9.13E-05 | 0.004602398 |
| Atp2b3 | ENSMUSG00000031376 | -0.515555026 | 9.34E-05 | 0.004659933 |
| Sgms1 | ENSMUSG00000040451 | 0.543724858 | 9.47E-05 | 0.004690634 |
| 1700020L24Rik | ENSMUSG00000035085 | 0.985106322 | 9.55E-05 | 0.004700371 |
| Nuak2 | ENSMUSG00000009772 | 0.98388696 | 9.68E-05 | 0.004713721 |
| Ncf2 | ENSMUSG00000026480 | 0.820770926 | 9.70E-05 | 0.004713721 |
| Foxn2 | ENSMUSG00000034998 | 0.5148333 | 9.76E-05 | 0.004713721 |
| Ptk2b | ENSMUSG00000059456 | -0.569601262 | 9.86E-05 | 0.004713721 |
| Slc16a4 | ENSMUSG00000027896 | 0.544403467 | 9.86E-05 | 0.004713721 |
| Itpripl2 | ENSMUSG00000095115 | 0.61001709 | 0.000100286 | 0.004760198 |
| Myct1 | ENSMUSG00000046916 | 0.70400999 | 0.000100541 | 0.004760198 |
| Klf15 | ENSMUSG00000030087 | 0.858584981 | 0.000110915 | 0.005134736 |
| Tm4sf1 | ENSMUSG00000027800 | 0.539540072 | 0.00011155 | 0.005134736 |
| Gjc3 | ENSMUSG00000056966 | 0.748964424 | 0.000112957 | 0.005181985 |
| 1700047M11Rik | ENSMUSG00000100147 | 0.745657186 | 0.000114211 | 0.005181985 |
| Efhd1 | ENSMUSG00000026255 | 0.938507864 | 0.000114314 | 0.005181985 |
| Tmem189 | ENSMUSG00000090213 | 0.59899675 | 0.000119718 | 0.005339354 |
| Tspan6 | ENSMUSG00000067377 | 0.501335174 | 0.00012023 | 0.005339354 |
| Car14 | ENSMUSG00000038526 | 0.89713163 | 0.000120569 | 0.005339354 |
| Ecel1 | ENSMUSG00000026247 | -1.526107553 | 0.000121971 | 0.005339354 |
| Rbpms | ENSMUSG00000031586 | 0.606309996 | 0.000122166 | 0.005339354 |
| Hfe | ENSMUSG00000006611 | 0.508544501 | 0.000123788 | 0.005370653 |
| Cdh4 | ENSMUSG00000000305 | -0.505178318 | 0.000126179 | 0.005423134 |
| Smim11 | ENSMUSG00000051989 | 0.512535308 | 0.000128372 | 0.005485784 |
| Tmem220 | ENSMUSG00000050270 | 0.935527839 | 0.000130628 | 0.005566229 |
| Vamp8 | ENSMUSG00000050732 | 0.640995529 | 0.000141592 | 0.005893112 |
| Slc25a45 | ENSMUSG00000024818 | 0.585753734 | 0.00014225 | 0.005893112 |
| Ccnd2 | ENSMUSG00000000184 | 0.57808264 | 0.000142865 | 0.005898684 |
| Higd1b | ENSMUSG00000020928 | 1.195670901 | 0.000143589 | 0.005899404 |
| Gm1043 | ENSMUSG00000117286 | -0.549654211 | 0.000145494 | 0.005928668 |
| Trf | ENSMUSG00000032554 | 1.186871165 | 0.000147371 | 0.005939286 |
| Tbxa2r | ENSMUSG00000034881 | 1.040378397 | 0.000151464 | 0.005995158 |
| Kcnj8 | ENSMUSG00000030247 | 1.047821481 | 0.000158501 | 0.006178709 |
| Galk1 | ENSMUSG00000020766 | 0.51845951 | 0.000164971 | 0.006357574 |
| Igfbp7 | ENSMUSG00000036256 | 0.590186314 | 0.00016831 | 0.006414108 |
| Nos3 | ENSMUSG00000028978 | 0.797141975 | 0.000168704 | 0.006414108 |
| Gal3st1 | ENSMUSG00000049721 | 0.932801753 | 0.000169019 | 0.006414108 |
| Ifi30 | ENSMUSG00000031838 | 0.674361846 | 0.000169488 | 0.006415595 |
| Kdm1b | ENSMUSG00000038080 | 0.550811069 | 0.000170079 | 0.006421683 |
| Evc2 | ENSMUSG00000050248 | 0.975629093 | 0.000172205 | 0.006469199 |
| Prr15 | ENSMUSG00000045725 | 1.19375092 | 0.000174665 | 0.006540356 |
| Ybx1 | ENSMUSG00000028639 | 0.648133476 | 0.000175177 | 0.006540356 |
| Ocln | ENSMUSG00000021638 | 0.798234419 | 0.000178409 | 0.006608547 |
| Tcim | ENSMUSG00000056313 | 0.595956711 | 0.000180742 | 0.006672258 |
| Klf2 | ENSMUSG00000055148 | 0.81602685 | 0.000181283 | 0.006675683 |
| Myo1a | ENSMUSG00000025401 | -0.535597947 | 0.000182792 | 0.006700919 |
| 2810468N07Rik | ENSMUSG00000091475 | 0.74209518 | 0.000184461 | 0.006742789 |
| Cnksr3 | ENSMUSG00000015202 | 0.585532938 | 0.000189485 | 0.00687587 |
| Elovl6 | ENSMUSG00000041220 | 0.586878591 | 0.000190293 | 0.006885387 |
| Scn11a | ENSMUSG00000034115 | -0.520960608 | 0.000196781 | 0.007021017 |
| AI506816 | ENSMUSG00000105987 | 0.616538014 | 0.00019908 | 0.007052446 |
| Ypel2 | ENSMUSG00000018427 | 0.601776662 | 0.000199677 | 0.007056822 |
| Fgd5 | ENSMUSG00000034037 | 0.531022953 | 0.000201325 | 0.007081926 |
| Chst2 | ENSMUSG00000033350 | 0.612261884 | 0.000201337 | 0.007081926 |
| Camk1g | ENSMUSG00000016179 | -0.533425753 | 0.000203538 | 0.00714251 |
| Wwtr1 | ENSMUSG00000027803 | 0.637467788 | 0.000209221 | 0.007261485 |
| Adamts4 | ENSMUSG00000006403 | 1.205455828 | 0.000209799 | 0.007261485 |
| Vstm4 | ENSMUSG00000050666 | 0.990671133 | 0.000211126 | 0.007282265 |
| Zfyve21 | ENSMUSG00000021286 | 0.61141925 | 0.000211427 | 0.007282265 |
| Arhgap29 | ENSMUSG00000039831 | 0.528878389 | 0.000219932 | 0.007488737 |
| Cd81 | ENSMUSG00000037706 | 0.569808982 | 0.00022977 | 0.007735415 |
| Rab12 | ENSMUSG00000023460 | 0.543573224 | 0.000230713 | 0.007742146 |
| Foxd3 | ENSMUSG00000067261 | 0.588519854 | 0.000231008 | 0.007742146 |
| Dynlt1-ps1 | ENSMUSG00000082691 | 0.636093019 | 0.000237282 | 0.007864042 |
| Gucy1b1 | ENSMUSG00000028005 | 0.558910696 | 0.000239243 | 0.00789395 |
| Mapkapk2 | ENSMUSG00000016528 | 0.559868372 | 0.00024042 | 0.007915277 |
| Mpp6 | ENSMUSG00000038388 | 0.630965321 | 0.000252056 | 0.008189907 |
| Tead3 | ENSMUSG00000002249 | 0.764277541 | 0.000256923 | 0.008328502 |
| Oaf | ENSMUSG00000032014 | 0.544681863 | 0.000260464 | 0.008371898 |
| Cavin1 | ENSMUSG00000004044 | 0.736612001 | 0.000263437 | 0.008393213 |
| Pcsk6 | ENSMUSG00000030513 | 0.982238481 | 0.000263941 | 0.008393213 |
| Sgk1 | ENSMUSG00000019970 | 0.832908105 | 0.000267515 | 0.008434935 |
| Kctd12 | ENSMUSG00000098557 | 0.667094147 | 0.000285216 | 0.008898964 |
| Pisd | ENSMUSG00000023452 | 0.60930569 | 0.00028774 | 0.008901818 |
| Adamts12 | ENSMUSG00000047497 | 0.83087722 | 0.000287756 | 0.008901818 |
| Gm37711 | ENSMUSG00000102796 | -0.567172345 | 0.000288607 | 0.008901818 |
| Tecta | ENSMUSG00000037705 | -0.503356311 | 0.000290082 | 0.008901818 |
| Fcgrt | ENSMUSG00000003420 | 0.534597682 | 0.000294126 | 0.008952223 |
| 4930429F24Rik | ENSMUSG00000086918 | 0.693927258 | 0.000297183 | 0.009008525 |
| Smox | ENSMUSG00000027333 | 0.501615466 | 0.00030668 | 0.009184398 |
| Tek | ENSMUSG00000006386 | 0.552579615 | 0.000322796 | 0.009514187 |
| Rnf32 | ENSMUSG00000029130 | -0.520068105 | 0.000323961 | 0.009519704 |
| Npepl1 | ENSMUSG00000039263 | 0.53670231 | 0.000329592 | 0.009638311 |
| Ado | ENSMUSG00000057134 | 0.583585805 | 0.000332654 | 0.00967547 |
| Ctdsp1 | ENSMUSG00000026176 | 0.566062795 | 0.000337117 | 0.009762655 |
| Rcn1 | ENSMUSG00000005973 | 0.622099214 | 0.000348097 | 0.009938948 |
| Apold1 | ENSMUSG00000090698 | 1.248895734 | 0.000355816 | 0.010031449 |
| mt-Nd2 | ENSMUSG00000064345 | 0.536096901 | 0.000357595 | 0.010043639 |
| Nid1 | ENSMUSG00000005397 | 0.701645711 | 0.000365351 | 0.01016811 |
| Map3k7cl | ENSMUSG00000025610 | 1.474987204 | 0.000367481 | 0.01016811 |
| Fndc3b | ENSMUSG00000039286 | 0.521599063 | 0.000372299 | 0.010244393 |
| Mxra8 | ENSMUSG00000029070 | 0.536011367 | 0.000373491 | 0.01025829 |
| Inppl1 | ENSMUSG00000032737 | 0.553928985 | 0.000390713 | 0.010575486 |
| Rilpl2 | ENSMUSG00000029401 | 0.583209651 | 0.00039537 | 0.010624422 |
| Vamp3 | ENSMUSG00000028955 | 0.55735791 | 0.000399845 | 0.010686892 |
| Ldlrad3 | ENSMUSG00000048058 | 0.571671562 | 0.00041509 | 0.010975294 |
| Pdgfrl | ENSMUSG00000031595 | 1.142288366 | 0.000415955 | 0.010975294 |
| Vamp5 | ENSMUSG00000073002 | 0.608670532 | 0.000416523 | 0.010975294 |
| Cavin2 | ENSMUSG00000045954 | 0.673167747 | 0.000420392 | 0.011057721 |
| Odc1 | ENSMUSG00000011179 | 0.533318003 | 0.000423785 | 0.011068876 |
| Ppm1m | ENSMUSG00000020253 | 0.528950668 | 0.000432032 | 0.011178267 |
| Dpm3 | ENSMUSG00000042737 | 0.552838089 | 0.000439195 | 0.011254548 |
| Slc5a11 | ENSMUSG00000030769 | -0.863145693 | 0.000443037 | 0.011333536 |
| St3gal3 | ENSMUSG00000028538 | 0.659058449 | 0.000446398 | 0.011380486 |
| Erbb4 | ENSMUSG00000062209 | 1.304078999 | 0.000450425 | 0.01144401 |
| Ppp1r16b | ENSMUSG00000037754 | 0.704822985 | 0.000453699 | 0.011488067 |
| Prob1 | ENSMUSG00000073600 | 0.919156734 | 0.000456896 | 0.011549409 |
| Il1rl2 | ENSMUSG00000070942 | 0.870435799 | 0.000458187 | 0.011556174 |
| Traf1 | ENSMUSG00000026875 | 0.717011008 | 0.000460184 | 0.011573669 |
| Pde5a | ENSMUSG00000053965 | 0.769203859 | 0.000467064 | 0.011695401 |
| Spin4 | ENSMUSG00000071722 | 1.027936317 | 0.000467377 | 0.011695401 |
| Retsat | ENSMUSG00000056666 | 0.532561414 | 0.000468634 | 0.011707222 |
| Csmd2 | ENSMUSG00000028804 | -0.553333932 | 0.000473011 | 0.01171803 |
| Tiam1 | ENSMUSG00000002489 | 0.512697357 | 0.000474482 | 0.01171803 |
| Plat | ENSMUSG00000031538 | 0.588033391 | 0.000475886 | 0.011731184 |
| A430005L14Rik | ENSMUSG00000047613 | 0.510135654 | 0.000481852 | 0.011842031 |
| Fhl3 | ENSMUSG00000032643 | 0.833712914 | 0.000483723 | 0.011842296 |
| Prkd3 | ENSMUSG00000024070 | 0.580411077 | 0.000486256 | 0.01186043 |
| F13a1 | ENSMUSG00000039109 | 0.575729177 | 0.000491113 | 0.011929095 |
| Map3k9 | ENSMUSG00000042724 | -0.523880902 | 0.000495302 | 0.011972352 |
| Tpm3 | ENSMUSG00000027940 | 0.672010503 | 0.00049785 | 0.012014458 |
| Zfp276 | ENSMUSG00000001065 | 0.617187157 | 0.000503446 | 0.012031216 |
| Ramp1 | ENSMUSG00000034353 | 1.389893771 | 0.000503674 | 0.012031216 |
| Smo | ENSMUSG00000001761 | 0.61058518 | 0.00051342 | 0.012192901 |
| Rftn2 | ENSMUSG00000025978 | 0.514163045 | 0.000517748 | 0.012256656 |
| Rab7b | ENSMUSG00000052688 | 0.684870984 | 0.000530067 | 0.012449475 |
| Metrn | ENSMUSG00000002274 | 0.686813518 | 0.000537669 | 0.012548966 |
| Xrcc4 | ENSMUSG00000021615 | 0.532157372 | 0.000544087 | 0.012659158 |
| Prickle1 | ENSMUSG00000036158 | 0.552694713 | 0.000546911 | 0.012705031 |
| Agpat2 | ENSMUSG00000026922 | 0.67146622 | 0.000556049 | 0.012897223 |
| Igf1 | ENSMUSG00000020053 | 0.727216165 | 0.000561826 | 0.012970708 |
| Col9a2 | ENSMUSG00000028626 | 0.833175446 | 0.000563919 | 0.012973762 |
| Flrt1 | ENSMUSG00000047787 | -0.54210827 | 0.000575559 | 0.013133031 |
| Kctd12b | ENSMUSG00000041633 | 0.536369157 | 0.000576783 | 0.013133031 |
| Tsc22d4 | ENSMUSG00000029723 | 0.610310094 | 0.000582867 | 0.013211071 |
| Rgs20 | ENSMUSG00000002459 | -0.696976039 | 0.000589703 | 0.013285238 |
| Apobec1 | ENSMUSG00000040613 | 0.588351708 | 0.000605423 | 0.013577862 |
| Syngr2 | ENSMUSG00000048277 | 0.608696193 | 0.000609109 | 0.013640017 |
| Phc1 | ENSMUSG00000040669 | 0.599475988 | 0.000612023 | 0.013684725 |
| Atf3 | ENSMUSG00000026628 | -0.826929538 | 0.000616571 | 0.013754714 |
| Lamc1 | ENSMUSG00000026478 | 0.681861473 | 0.000635251 | 0.014035744 |
| Pecam1 | ENSMUSG00000020717 | 0.611931058 | 0.000643393 | 0.014173655 |
| A330102I10Rik | ENSMUSG00000086363 | -0.547699877 | 0.000675225 | 0.014701183 |
| Txnip | ENSMUSG00000038393 | 0.545790696 | 0.000682411 | 0.014749975 |
| Kif1c | ENSMUSG00000020821 | 0.665299291 | 0.000684092 | 0.014753882 |
| Aoc3 | ENSMUSG00000019326 | 0.593572472 | 0.000699481 | 0.014988594 |
| Sh3bp5 | ENSMUSG00000021892 | 0.659022432 | 0.000706399 | 0.015115113 |
| 1810037I17Rik | ENSMUSG00000054091 | 0.510025136 | 0.000711268 | 0.015163223 |
| Adh1 | ENSMUSG00000074207 | 0.901825992 | 0.00073799 | 0.015589771 |
| Gng5 | ENSMUSG00000068523 | 0.568899006 | 0.000752681 | 0.015721968 |
| Snta1 | ENSMUSG00000027488 | 0.655726822 | 0.00077855 | 0.016126797 |
| Ccdc60 | ENSMUSG00000043913 | -0.941146718 | 0.00078532 | 0.016244464 |
| Trim13 | ENSMUSG00000035235 | 0.537816453 | 0.000788743 | 0.01629268 |
| Lamb1 | ENSMUSG00000002900 | 0.666171203 | 0.000796618 | 0.016342174 |
| Rcn3 | ENSMUSG00000019539 | 0.727065211 | 0.000801698 | 0.016401274 |
| Fgfr3 | ENSMUSG00000054252 | 1.278091731 | 0.000805015 | 0.016424063 |
| Dmd | ENSMUSG00000045103 | 0.511219473 | 0.000807561 | 0.016453506 |
| C2cd4d | ENSMUSG00000091648 | 1.034382287 | 0.000822263 | 0.016616852 |
| Tfeb | ENSMUSG00000023990 | 0.553320648 | 0.000835065 | 0.01673946 |
| Cnp | ENSMUSG00000006782 | 0.713133752 | 0.000839118 | 0.016762326 |
| Samd4 | ENSMUSG00000021838 | 0.599572107 | 0.000847867 | 0.016863134 |
| St6gal1 | ENSMUSG00000022885 | 0.596514221 | 0.000852826 | 0.016936154 |
| Sowaha | ENSMUSG00000044352 | 0.595412654 | 0.000854175 | 0.016940376 |
| Yap1 | ENSMUSG00000053110 | 0.582134857 | 0.000857815 | 0.016959762 |
| Idh2 | ENSMUSG00000030541 | 0.671275724 | 0.000858564 | 0.016959762 |
| Metrnl | ENSMUSG00000039208 | 0.859887484 | 0.000869722 | 0.017134793 |
| Slc41a1 | ENSMUSG00000013275 | 0.562518166 | 0.000871008 | 0.017137493 |
| Ace | ENSMUSG00000020681 | 0.598744438 | 0.000880844 | 0.017262684 |
| Pros1 | ENSMUSG00000022912 | 0.536824126 | 0.000900591 | 0.01758039 |
| Ttc39aos1 | ENSMUSG00000085873 | -0.5500334 | 0.00091327 | 0.017758151 |
| Col9a1 | ENSMUSG00000026147 | 4.266983408 | 0.000932501 | 0.01806145 |
| Gm43667 | ENSMUSG00000105135 | 0.593270822 | 0.000952857 | 0.018313034 |
| Mavs | ENSMUSG00000037523 | 0.585373731 | 0.000955393 | 0.018338134 |
| Spry1 | ENSMUSG00000037211 | 0.829231742 | 0.000978835 | 0.018739847 |
| Kank2 | ENSMUSG00000032194 | 0.548963862 | 0.000980385 | 0.018745455 |
| Crybb1 | ENSMUSG00000029343 | 0.806721674 | 0.000998936 | 0.018858395 |
| Stk3 | ENSMUSG00000022329 | 0.531465315 | 0.001006112 | 0.018936287 |
| Qk | ENSMUSG00000062078 | 0.724207271 | 0.001033712 | 0.019175105 |
| Tgfbr2 | ENSMUSG00000032440 | 0.529070109 | 0.001035984 | 0.019193373 |
| Mob3b | ENSMUSG00000073910 | 0.734697504 | 0.001053893 | 0.019335926 |
| Hdx | ENSMUSG00000034551 | -0.528426322 | 0.001058814 | 0.019393329 |
| Gm42047 | ENSMUSG00000110631 | 0.977543468 | 0.00105978 | 0.019393329 |
| Hmgn5 | ENSMUSG00000031245 | 0.814685149 | 0.001064589 | 0.019457452 |
| Slfn5 | ENSMUSG00000054404 | 0.869347727 | 0.00106815 | 0.019498633 |
| Mfge8 | ENSMUSG00000030605 | 0.753638193 | 0.001084561 | 0.019670801 |
| Zcchc24 | ENSMUSG00000055538 | 0.542834879 | 0.001084799 | 0.019670801 |
| Selenop | ENSMUSG00000064373 | 0.611224182 | 0.001088119 | 0.019694433 |
| Smad3 | ENSMUSG00000032402 | 0.681219224 | 0.001096512 | 0.019753527 |
| Acy3 | ENSMUSG00000024866 | 0.708057369 | 0.001127079 | 0.020058786 |
| Rtl5 | ENSMUSG00000049191 | 0.507962406 | 0.001134768 | 0.020171549 |
| P4ha1 | ENSMUSG00000019916 | 0.601940609 | 0.001136556 | 0.020179288 |
| Cers2 | ENSMUSG00000015714 | 0.664669655 | 0.001144838 | 0.020253983 |
| Prr7 | ENSMUSG00000034686 | 0.75040627 | 0.001155366 | 0.020326599 |
| Cx3cl1 | ENSMUSG00000031778 | 0.738520203 | 0.001163311 | 0.02038733 |
| Lzts2 | ENSMUSG00000035342 | 0.629825422 | 0.001187194 | 0.020636139 |
| Fbln7 | ENSMUSG00000027386 | 0.946007739 | 0.001188581 | 0.020636204 |
| Evc | ENSMUSG00000029122 | 0.577020263 | 0.001200733 | 0.020751096 |
| Npnt | ENSMUSG00000040998 | 0.946367525 | 0.001206652 | 0.020791789 |
| Usp6nl | ENSMUSG00000039046 | 0.622068576 | 0.001207301 | 0.020791789 |
| Myrfl | ENSMUSG00000034057 | 0.870222551 | 0.001220628 | 0.020972865 |
| Pdgfd | ENSMUSG00000032006 | 0.549143426 | 0.001233225 | 0.021116316 |
| Prss23 | ENSMUSG00000039405 | 0.562228303 | 0.001248312 | 0.021301281 |
| Gltp | ENSMUSG00000011884 | 0.527808123 | 0.001249785 | 0.021302054 |
| Amotl2 | ENSMUSG00000032531 | 0.604782384 | 0.001265629 | 0.021522911 |
| Syde2 | ENSMUSG00000036863 | 0.6671506 | 0.001268643 | 0.021549584 |
| Ppp1r26 | ENSMUSG00000035829 | 0.552415116 | 0.001283541 | 0.021661135 |
| Castor1 | ENSMUSG00000020424 | 0.542103775 | 0.001293525 | 0.021798448 |
| Sox9 | ENSMUSG00000000567 | 1.108419726 | 0.001312295 | 0.021929517 |
| Triobp | ENSMUSG00000033088 | 0.589044082 | 0.00131861 | 0.021977474 |
| Klf4 | ENSMUSG00000003032 | 0.791552131 | 0.001318884 | 0.021977474 |
| Pdlim2 | ENSMUSG00000022090 | 0.522060723 | 0.001325852 | 0.022044324 |
| Cmtm5 | ENSMUSG00000040759 | 0.612633287 | 0.001332328 | 0.022127331 |
| Cobll1 | ENSMUSG00000034903 | 0.545794397 | 0.001337156 | 0.022182807 |
| 4930523C07Rik | ENSMUSG00000090394 | 0.742149658 | 0.001345632 | 0.022298623 |
| Rab29 | ENSMUSG00000026433 | 0.525568862 | 0.001351955 | 0.022378524 |
| Pcolce | ENSMUSG00000029718 | 0.754622947 | 0.001357774 | 0.022425073 |
| Plin3 | ENSMUSG00000024197 | 0.576688958 | 0.001364208 | 0.022506421 |
| Arhgap10 | ENSMUSG00000037148 | 0.654337025 | 0.001385695 | 0.022810442 |
| Fgfbp3 | ENSMUSG00000047632 | 0.812825148 | 0.00139485 | 0.022935831 |
| Serhl | ENSMUSG00000058586 | 0.625254525 | 0.001409067 | 0.023101864 |
| Ttll6 | ENSMUSG00000038756 | 0.791250527 | 0.001410673 | 0.023101864 |
| Tmem88 | ENSMUSG00000045377 | 0.733274616 | 0.001411144 | 0.023101864 |
| Adamtsl2 | ENSMUSG00000036040 | 0.752152901 | 0.001430593 | 0.023321545 |
| Jam3 | ENSMUSG00000031990 | 0.702410298 | 0.001440083 | 0.023395854 |
| Samd5 | ENSMUSG00000060487 | 0.598976478 | 0.001446054 | 0.023420302 |
| Zfp395 | ENSMUSG00000034522 | 0.571588046 | 0.001462281 | 0.023627792 |
| Mras | ENSMUSG00000032470 | 0.619360479 | 0.00146918 | 0.0237049 |
| Agmo | ENSMUSG00000050103 | 0.520407041 | 0.001470231 | 0.0237049 |
| Plekhg5 | ENSMUSG00000039713 | 0.538592115 | 0.00150393 | 0.024066102 |
| Dhrs3 | ENSMUSG00000066026 | 0.714364993 | 0.001506992 | 0.024089264 |
| Nkd1 | ENSMUSG00000031661 | 0.700270188 | 0.001519898 | 0.02423964 |
| L3mbtl4 | ENSMUSG00000041565 | -0.588495341 | 0.001521909 | 0.02423964 |
| F9 | ENSMUSG00000031138 | -0.587475382 | 0.001526752 | 0.02423964 |
| Gja1 | ENSMUSG00000050953 | 0.617260943 | 0.001531027 | 0.02423964 |
| Slc36a1os | ENSMUSG00000084980 | 0.987867538 | 0.001533579 | 0.024254296 |
| Lrrtm4 | ENSMUSG00000052581 | 0.940127021 | 0.001557141 | 0.024522915 |
| Wtip | ENSMUSG00000036459 | 0.588296686 | 0.001562257 | 0.024577527 |
| Bcl6b | ENSMUSG00000000317 | 0.969176244 | 0.001564838 | 0.0245922 |
| Cav1 | ENSMUSG00000007655 | 0.659375952 | 0.001574913 | 0.024724471 |
| Tram2 | ENSMUSG00000041779 | 0.700586995 | 0.001589997 | 0.024879768 |
| Crygs | ENSMUSG00000033501 | 1.065776063 | 0.001594291 | 0.024879768 |
| Stard8 | ENSMUSG00000031216 | 0.659436949 | 0.001597648 | 0.024879768 |
| Art4 | ENSMUSG00000030217 | 0.753358699 | 0.001605318 | 0.02493929 |
| Itga2b | ENSMUSG00000034664 | 0.638500404 | 0.001609605 | 0.024957814 |
| Cytl1 | ENSMUSG00000062329 | 3.620244206 | 0.001610774 | 0.024957814 |
| Cxcl14 | ENSMUSG00000021508 | 1.072799616 | 0.001611531 | 0.024957814 |
| Trim47 | ENSMUSG00000020773 | 0.711368784 | 0.001643905 | 0.025301541 |
| Plekhf1 | ENSMUSG00000074170 | 0.893223945 | 0.001655556 | 0.025376116 |
| Ifit3 | ENSMUSG00000074896 | 0.772854158 | 0.001661749 | 0.025418788 |
| Cmtm3 | ENSMUSG00000031875 | 0.5024993 | 0.001683566 | 0.025542837 |
| Plp1 | ENSMUSG00000031425 | 0.6130278 | 0.001686985 | 0.025542837 |
| Cd68 | ENSMUSG00000018774 | 0.923164993 | 0.001692294 | 0.025597233 |
| 1810014B01Rik | ENSMUSG00000097412 | 0.835289453 | 0.001697745 | 0.025632723 |
| Tmem181b-ps | ENSMUSG00000096780 | 0.695052499 | 0.001703263 | 0.025632723 |
| Erg | ENSMUSG00000040732 | 0.618353197 | 0.001716232 | 0.025633575 |
| Atp8b1 | ENSMUSG00000039529 | 0.583602599 | 0.0017181 | 0.025633575 |
| Btbd17 | ENSMUSG00000000202 | 1.232369931 | 0.001755655 | 0.026027676 |
| Enpp2 | ENSMUSG00000022425 | 0.75315766 | 0.001763741 | 0.026043999 |
| Snhg18 | ENSMUSG00000096956 | 0.787200962 | 0.001769647 | 0.02605785 |
| Il6ra | ENSMUSG00000027947 | 0.55292807 | 0.001786915 | 0.026179567 |
| Bmp1 | ENSMUSG00000022098 | 0.547588373 | 0.001786965 | 0.026179567 |
| Celf2 | ENSMUSG00000002107 | 0.583613399 | 0.001792605 | 0.026210354 |
| H2-DMa | ENSMUSG00000037649 | 0.728438248 | 0.001833116 | 0.026568601 |
| Capn6 | ENSMUSG00000067276 | 0.743117927 | 0.001839198 | 0.026605038 |
| Insrr | ENSMUSG00000005640 | -0.546346649 | 0.001856207 | 0.026825074 |
| Evi2a | ENSMUSG00000078771 | 0.853978964 | 0.001866219 | 0.026865621 |
| Pttg1ip | ENSMUSG00000009291 | 0.568128245 | 0.001876517 | 0.026961823 |
| Ltbp4 | ENSMUSG00000040488 | 0.506218979 | 0.001887328 | 0.027090143 |
| AC129186.1 | ENSMUSG00000116610 | -0.577911313 | 0.001890897 | 0.027090143 |
| Tox3 | ENSMUSG00000043668 | -0.508580171 | 0.001898236 | 0.027101048 |
| Rab40b | ENSMUSG00000025170 | 0.831063842 | 0.001903896 | 0.027120058 |
| Myl3 | ENSMUSG00000059741 | 3.38927915 | 0.001957304 | 0.027617065 |
| Tll1 | ENSMUSG00000053626 | 0.6398642 | 0.001988172 | 0.027815757 |
| Gpm6a | ENSMUSG00000031517 | 0.611074584 | 0.001995303 | 0.027863247 |
| Mpz | ENSMUSG00000056569 | 1.194370157 | 0.001999581 | 0.027880481 |
| Nfix | ENSMUSG00000001911 | 0.671506076 | 0.002001771 | 0.027880481 |
| Gm28578 | ENSMUSG00000100417 | -1.117952715 | 0.002014839 | 0.028004945 |
| Cdc42ep1 | ENSMUSG00000049521 | 0.708738312 | 0.002039333 | 0.028266375 |
| Apol6 | ENSMUSG00000033576 | 1.665190216 | 0.002058651 | 0.02840215 |
| Sipa1 | ENSMUSG00000056917 | 0.545348851 | 0.002063322 | 0.028440277 |
| Mt1 | ENSMUSG00000031765 | 0.655525897 | 0.00206768 | 0.028462218 |
| Lmcd1 | ENSMUSG00000057604 | 1.134882576 | 0.00206873 | 0.028462218 |
| Six5 | ENSMUSG00000040841 | 0.652138105 | 0.002075338 | 0.028500538 |
| Gpd1 | ENSMUSG00000023019 | 0.621466996 | 0.002113266 | 0.028884069 |
| Vwf | ENSMUSG00000001930 | 0.500432927 | 0.002118909 | 0.028884069 |
| Hotair | ENSMUSG00000086903 | 1.622057962 | 0.002120696 | 0.028884069 |
| Tmbim1 | ENSMUSG00000006301 | 0.528123745 | 0.002125124 | 0.028898143 |
| Lrrc8e | ENSMUSG00000046589 | 1.056803553 | 0.002127542 | 0.028898143 |
| Cyp2j13 | ENSMUSG00000028571 | 0.877368616 | 0.002136306 | 0.028964424 |
| Slc12a4 | ENSMUSG00000017765 | 0.527867376 | 0.00215034 | 0.0290368 |
| Net1 | ENSMUSG00000021215 | 0.513707361 | 0.002155207 | 0.0290368 |
| Dlg1 | ENSMUSG00000022770 | 0.512606995 | 0.002158031 | 0.029047716 |
| Foxo4 | ENSMUSG00000042903 | 0.603740207 | 0.002160613 | 0.029056248 |
| Ly6e | ENSMUSG00000022587 | 0.634227111 | 0.00216528 | 0.029066595 |
| Rnf39 | ENSMUSG00000036492 | -0.887255098 | 0.002181275 | 0.029202454 |
| Osbpl5 | ENSMUSG00000037606 | 0.552521999 | 0.002195506 | 0.029318064 |
| Rp1 | ENSMUSG00000025900 | 0.944571043 | 0.002195808 | 0.029318064 |
| Megf6 | ENSMUSG00000057751 | 0.653969233 | 0.00222136 | 0.029553404 |
| Lhpp | ENSMUSG00000030946 | 0.578480739 | 0.002224315 | 0.029566346 |
| Tmem200c | ENSMUSG00000095407 | -0.540926127 | 0.002236549 | 0.029676066 |
| Copz2 | ENSMUSG00000018672 | 0.648963621 | 0.002247855 | 0.02974668 |
| Gramd3 | ENSMUSG00000001700 | 0.584411902 | 0.002258614 | 0.029836107 |
| Dynlt1b | ENSMUSG00000096255 | 0.680238413 | 0.002320289 | 0.030248939 |
| Loxl3 | ENSMUSG00000000693 | 0.540558449 | 0.002327752 | 0.030312099 |
| Flvcr2 | ENSMUSG00000034258 | 0.583447522 | 0.002338549 | 0.030357975 |
| Col4a1 | ENSMUSG00000031502 | 0.953702517 | 0.002357422 | 0.030493143 |
| Olfr558 | ENSMUSG00000070423 | 0.788523014 | 0.00236424 | 0.030525626 |
| Gm11361 | ENSMUSG00000113061 | 1.062833252 | 0.002379207 | 0.030589218 |
| Myoz2 | ENSMUSG00000028116 | 2.777360893 | 0.002431196 | 0.030953742 |
| Pcdhga1 | ENSMUSG00000103144 | -0.739896341 | 0.002432465 | 0.030953742 |
| Enpp6 | ENSMUSG00000038173 | 1.430849651 | 0.002437082 | 0.030986055 |
| Nupr1 | ENSMUSG00000030717 | 1.021335617 | 0.002440995 | 0.031009371 |
| Gcnt1 | ENSMUSG00000038843 | 0.646538479 | 0.002505147 | 0.031555544 |
| Cdt1 | ENSMUSG00000006585 | 0.946620136 | 0.002525821 | 0.031789112 |
| Hbegf | ENSMUSG00000024486 | 0.680614008 | 0.002596392 | 0.032379633 |
| Csf1r | ENSMUSG00000024621 | 0.602464915 | 0.002615213 | 0.032475672 |
| Olfml1 | ENSMUSG00000051041 | 0.697598177 | 0.002617843 | 0.032481286 |
| Rai14 | ENSMUSG00000022246 | 0.502176518 | 0.002626477 | 0.032534281 |
| Tnni1 | ENSMUSG00000026418 | 2.672455502 | 0.002674433 | 0.03293682 |
| Fli1 | ENSMUSG00000016087 | 0.547366825 | 0.002683841 | 0.03302542 |
| Col3a1 | ENSMUSG00000026043 | 1.074672338 | 0.002686987 | 0.033036866 |
| Nde1 | ENSMUSG00000022678 | 0.530080232 | 0.002711666 | 0.033176196 |
| Pdgfc | ENSMUSG00000028019 | 0.556977108 | 0.002730293 | 0.033316871 |
| Tead4 | ENSMUSG00000030353 | 0.764144984 | 0.002733533 | 0.033316871 |
| Hsd11b1 | ENSMUSG00000016194 | 0.859398878 | 0.002744164 | 0.033357426 |
| Carns1 | ENSMUSG00000075289 | 0.619410554 | 0.002773605 | 0.033612209 |
| Eva1b | ENSMUSG00000050212 | 0.817446835 | 0.002809344 | 0.033953452 |
| Stard5 | ENSMUSG00000046027 | 0.676984782 | 0.002824446 | 0.034108332 |
| Cryab | ENSMUSG00000032060 | 0.953336724 | 0.002827379 | 0.034116121 |
| Itgav | ENSMUSG00000027087 | 0.53442598 | 0.002842351 | 0.034269053 |
| Hepacam | ENSMUSG00000046240 | 0.65830876 | 0.002849973 | 0.034298067 |
| Meox1 | ENSMUSG00000001493 | 1.055922888 | 0.002859432 | 0.034317376 |
| Caskin2 | ENSMUSG00000034471 | 0.61937506 | 0.002871302 | 0.034340499 |
| Col4a2 | ENSMUSG00000031503 | 0.958074686 | 0.00289543 | 0.034435762 |
| Tnnc1 | ENSMUSG00000091898 | 3.013159271 | 0.002927387 | 0.034705127 |
| Tjp2 | ENSMUSG00000024812 | 0.651831521 | 0.002934945 | 0.034711956 |
| Lrriq1 | ENSMUSG00000019892 | -0.751545957 | 0.002980336 | 0.03510958 |
| Cryzl2 | ENSMUSG00000033488 | 0.743903528 | 0.003002412 | 0.035230507 |
| Tpm3-rs7 | ENSMUSG00000058126 | 0.682385531 | 0.003021385 | 0.035376565 |
| Gstm2 | ENSMUSG00000040562 | 0.539812779 | 0.003043995 | 0.035500783 |
| Stum | ENSMUSG00000053963 | 0.660551085 | 0.003048032 | 0.035514336 |
| Gm16334 | ENSMUSG00000089757 | -0.632973956 | 0.003060735 | 0.035634502 |
| Peli2 | ENSMUSG00000021846 | 0.503753736 | 0.003064526 | 0.035644622 |
| Emp2 | ENSMUSG00000022505 | 0.65312844 | 0.003091171 | 0.035793259 |
| Fut8 | ENSMUSG00000021065 | 0.582002308 | 0.003095857 | 0.035819708 |
| Gkn3 | ENSMUSG00000030048 | 0.89847437 | 0.003149176 | 0.036352826 |
| Nrep | ENSMUSG00000042834 | 0.635039265 | 0.003149246 | 0.036352826 |
| Tmem40 | ENSMUSG00000059900 | 0.909396314 | 0.0031825 | 0.036579673 |
| Grap | ENSMUSG00000004837 | 0.566702234 | 0.003183614 | 0.036579673 |
| A730049H05Rik | ENSMUSG00000048636 | 0.89427891 | 0.003198686 | 0.036656011 |
| Zfp185 | ENSMUSG00000031351 | 0.841029958 | 0.003206845 | 0.036656011 |
| Tnnt1 | ENSMUSG00000064179 | 3.621879989 | 0.0032179 | 0.036719026 |
| Fam83f | ENSMUSG00000022408 | 0.702855666 | 0.00323118 | 0.036807331 |
| Tead1 | ENSMUSG00000055320 | 0.606422549 | 0.003240947 | 0.036841073 |
| Apol11b | ENSMUSG00000091694 | -1.676826578 | 0.003262602 | 0.036974503 |
| Depp1 | ENSMUSG00000048489 | 0.867166268 | 0.003303334 | 0.037193756 |
| Rasgef1c | ENSMUSG00000020374 | 0.726333326 | 0.003306158 | 0.037193756 |
| Ifitm3 | ENSMUSG00000025492 | 0.592070276 | 0.003315486 | 0.037193756 |
| Fa2h | ENSMUSG00000033579 | 1.149739458 | 0.003318727 | 0.037193756 |
| Slc25a43 | ENSMUSG00000037636 | -0.80275725 | 0.00332328 | 0.037193756 |
| Cbr2 | ENSMUSG00000025150 | 0.81813514 | 0.003324345 | 0.037193756 |
| Chpt1 | ENSMUSG00000060002 | 0.524489239 | 0.003334033 | 0.03727419 |
| Raver2 | ENSMUSG00000035275 | 0.740941636 | 0.003342227 | 0.037297043 |
| C1qa | ENSMUSG00000036887 | 0.584068211 | 0.003354254 | 0.037360227 |
| Bhlhe22 | ENSMUSG00000025128 | 1.141736216 | 0.00336311 | 0.037403004 |
| Sema4c | ENSMUSG00000026121 | 0.570397395 | 0.003379263 | 0.037526675 |
| Cdh5 | ENSMUSG00000031871 | 0.561015147 | 0.003385924 | 0.037540534 |
| Adam10 | ENSMUSG00000054693 | 0.650747654 | 0.00343918 | 0.037965902 |
| Tmem266 | ENSMUSG00000032313 | -0.732953311 | 0.003461821 | 0.038074929 |
| Fzd4 | ENSMUSG00000049791 | 0.501510801 | 0.003469293 | 0.038085504 |
| Adrb2 | ENSMUSG00000045730 | 0.667960038 | 0.003474089 | 0.038085504 |
| Pxdc1 | ENSMUSG00000021411 | 0.734890215 | 0.003511378 | 0.038281205 |
| Igf2bp2 | ENSMUSG00000033581 | 0.781606916 | 0.003603857 | 0.038891408 |
| Ltbp1 | ENSMUSG00000001870 | 0.723387615 | 0.003611825 | 0.038949214 |
| Scrib | ENSMUSG00000022568 | 0.50526883 | 0.003663198 | 0.039237528 |
| Gpm6b | ENSMUSG00000031342 | 0.535953097 | 0.003687639 | 0.039446932 |
| Tor3a | ENSMUSG00000060519 | 0.535457584 | 0.003745901 | 0.039906912 |
| Slc16a13 | ENSMUSG00000044367 | 0.632940025 | 0.003749904 | 0.039918677 |
| Ccdc13 | ENSMUSG00000079235 | 0.745522851 | 0.003778362 | 0.04010711 |
| Cdkn1a | ENSMUSG00000023067 | 0.657073412 | 0.003803854 | 0.040137186 |
| Pmp22 | ENSMUSG00000018217 | 1.092337952 | 0.00381098 | 0.040137186 |
| Ldlrap1 | ENSMUSG00000037295 | 0.688027094 | 0.003814347 | 0.040137186 |
| Sh3tc2 | ENSMUSG00000045629 | 0.716286406 | 0.003814448 | 0.040137186 |
| Tnmd | ENSMUSG00000031250 | 3.324417272 | 0.003814718 | 0.040137186 |
| Tril | ENSMUSG00000043496 | 0.541401065 | 0.003831644 | 0.040243057 |
| Syt17 | ENSMUSG00000058420 | -0.86382884 | 0.003839344 | 0.040295548 |
| Itm2a | ENSMUSG00000031239 | 0.916733916 | 0.003859367 | 0.040477211 |
| Mfsd2a | ENSMUSG00000028655 | 0.519792017 | 0.003880237 | 0.040638943 |
| Cav2 | ENSMUSG00000000058 | 0.569391103 | 0.003904018 | 0.040773472 |
| Hspb2 | ENSMUSG00000038086 | 1.072512112 | 0.003936353 | 0.040996343 |
| 4930516B21Rik | ENSMUSG00000097504 | 0.672190997 | 0.00398419 | 0.04135018 |
| C1qtnf9 | ENSMUSG00000071347 | 1.402423809 | 0.004002955 | 0.041487198 |
| Ttyh2 | ENSMUSG00000034714 | 0.62128236 | 0.004017497 | 0.041580116 |
| Mvb12b | ENSMUSG00000038740 | 0.539440733 | 0.004029441 | 0.041646043 |
| Fbxl8 | ENSMUSG00000033313 | -0.642943195 | 0.004029452 | 0.041646043 |
| Cubn | ENSMUSG00000026726 | -0.593756576 | 0.004074623 | 0.041909606 |
| Gm31812 | ENSMUSG00000109599 | 1.0650491 | 0.004092696 | 0.041979005 |
| Frzb | ENSMUSG00000027004 | 0.680613284 | 0.004112751 | 0.042098536 |
| Ugt8a | ENSMUSG00000032854 | 1.243061792 | 0.004123803 | 0.042104908 |
| Zfp786 | ENSMUSG00000051499 | -0.726450787 | 0.004174131 | 0.042464526 |
| Pcdha12 | ENSMUSG00000103310 | -0.535863687 | 0.004187512 | 0.042542617 |
| Sod3 | ENSMUSG00000072941 | 0.510512744 | 0.004281812 | 0.043089372 |
| Wif1 | ENSMUSG00000020218 | 3.905948567 | 0.004294064 | 0.043089372 |
| Gm37226 | ENSMUSG00000102533 | -0.663583076 | 0.00430116 | 0.043089372 |
| Pcdha11 | ENSMUSG00000007440 | -0.874153575 | 0.004301904 | 0.043089372 |
| Phldb1 | ENSMUSG00000048537 | 0.53573455 | 0.004302003 | 0.043089372 |
| Col2a1 | ENSMUSG00000022483 | 4.147335972 | 0.004325127 | 0.043251134 |
| Myl2 | ENSMUSG00000013936 | 3.103165002 | 0.004329033 | 0.043251134 |
| Pkdcc | ENSMUSG00000024247 | 0.614319735 | 0.004358022 | 0.043368017 |
| Sgcz | ENSMUSG00000039539 | -0.701646789 | 0.004383635 | 0.043498024 |
| Pea15a | ENSMUSG00000013698 | 0.506722644 | 0.0044107 | 0.043737491 |
| Lrig1 | ENSMUSG00000030029 | 0.535916854 | 0.0044367 | 0.043836898 |
| Serpina1e | ENSMUSG00000072849 | -0.70369184 | 0.0044413 | 0.043836898 |
| Pla2g5 | ENSMUSG00000041193 | 0.9412938 | 0.004478849 | 0.044097028 |
| Fscn1 | ENSMUSG00000029581 | 1.027970788 | 0.004517801 | 0.044315269 |
| Dll4 | ENSMUSG00000027314 | 0.52680637 | 0.00451948 | 0.044315269 |
| Il17rb | ENSMUSG00000015966 | 0.756145703 | 0.004568914 | 0.044711802 |
| Pdk4 | ENSMUSG00000019577 | 1.596130462 | 0.004643443 | 0.045262943 |
| Plekhg2 | ENSMUSG00000037552 | 0.654094805 | 0.004654987 | 0.04531624 |
| St8sia5 | ENSMUSG00000025425 | 0.717069758 | 0.004684436 | 0.045470127 |
| Magee2 | ENSMUSG00000031224 | -0.637412465 | 0.004694562 | 0.045493627 |
| Sox18 | ENSMUSG00000046470 | 0.66936102 | 0.004726861 | 0.045687096 |
| Clec14a | ENSMUSG00000045930 | 0.810132858 | 0.004729843 | 0.045687096 |
| Fkbp7 | ENSMUSG00000002732 | 0.587644744 | 0.004764373 | 0.045901715 |
| Itih3 | ENSMUSG00000006522 | 1.529766792 | 0.004811169 | 0.046143908 |
| Gm31166 | ENSMUSG00000109695 | 0.821722489 | 0.004817681 | 0.046176667 |
| Pou3f1 | ENSMUSG00000090125 | 0.552177086 | 0.004824135 | 0.046208831 |
| Ndrg1 | ENSMUSG00000005125 | 0.6441031 | 0.004834886 | 0.046229699 |
| Col1a2 | ENSMUSG00000029661 | 1.522544904 | 0.004870237 | 0.046309801 |
| Spint1 | ENSMUSG00000027315 | 0.723299286 | 0.004883566 | 0.046337547 |
| Fam205c | ENSMUSG00000050141 | -0.585912649 | 0.00488768 | 0.046341298 |
| Dusp14 | ENSMUSG00000018648 | 0.635179965 | 0.004896224 | 0.046369012 |
| Clec2d | ENSMUSG00000030157 | 0.727108414 | 0.00491338 | 0.046377757 |
| Mal | ENSMUSG00000027375 | 1.058624022 | 0.004916455 | 0.046377757 |
| Swap70 | ENSMUSG00000031015 | 0.55468755 | 0.004943928 | 0.046549078 |
| Miat | ENSMUSG00000097767 | -0.667363588 | 0.005019689 | 0.047054462 |
| Cilp2 | ENSMUSG00000044006 | 2.701441807 | 0.005057402 | 0.047289091 |
| Slc20a2 | ENSMUSG00000037656 | 0.601776015 | 0.005214103 | 0.048372389 |
| C78859 | ENSMUSG00000110710 | -0.583180546 | 0.005240779 | 0.048567128 |
| Dpp4 | ENSMUSG00000035000 | 0.776323873 | 0.00527251 | 0.048720081 |
| Eln | ENSMUSG00000029675 | 1.303695495 | 0.005290911 | 0.04879941 |
| Rftn1 | ENSMUSG00000039316 | 0.892616391 | 0.005358015 | 0.049189103 |
| Cdc42ep3 | ENSMUSG00000036533 | 0.598093083 | 0.005359548 | 0.049189103 |
| Paqr6 | ENSMUSG00000041423 | 0.5066204 | 0.005401143 | 0.049509924 |
| Ndufa4l2 | ENSMUSG00000040280 | 0.962645524 | 0.005432761 | 0.049769164 |
| Ccdc102a | ENSMUSG00000063605 | 0.542076145 | 0.005447743 | 0.049784097 |

## Supplementary Table 2

| Luminex Multifactor Assay for Differentially Expressed Genes | | |
| --- | --- | --- |
|  | **Cytokine** | **p_value** |
| 1 | TNF-alpha | 7.87E-10 |
| 2 | GM-CSF | 1.22E-06 |
| 3 | TARC-CCL17 | 3.02E-05 |
| 4 | IL-6 | 1.07E-04 |
| 5 | Fractalkine-CX3CL1 | 2.94E-04 |
| 6 | MCP-1-CCL2 | 4.32E-02 |
| 7 | MIP-3alpha-CCL20 | 4.41E-02 |
| 8 | MDC-CCL22 | 4.46E-02 |
